# Epigenetic regulation of cancer stemness

**DOI:** 10.1038/s41392-025-02340-6

**Published:** 2025-08-01

**Authors:** Claudia Galassi, Gwenola Manic, Manel Esteller, Lorenzo Galluzzi, Ilio Vitale

**Affiliations:** 1 Department of Pharmacology, Weill Cornell Medical College, New York, NY USA; 2 Italian Institute for Genomic Medicine, c/o IRCSS Candiolo, Torino, Italy; 3 Candiolo Cancer Institute, FPO-IRCCS, Candiolo, Italy; 4 Cancer Epigenetics Group, Josep Carreras Leukemia Research Institute (IJC), Barcelona, Spain; 5 Centro de Investigacion Biomedica en Red Cancer (CIBERONC), Madrid, Spain; 6 Institució Catalana de Recerca i Estudis Avançats (ICREA), Barcelona, Spain; 7 Physiological Sciences Department, School of Medicine and Health Sciences, University of Barcelona (UB), Barcelona, Spain; 8 Cancer Signaling and Microenvironment Program, Fox Chase Cancer Center, Philadelphia, PA USA

**Keywords:** Cancer stem cells, Cancer therapy

## Abstract

Gene expression is finely controlled by the abundance and activation status of transcription factors and their regulators, as well as by a number of reversible modifications of DNA and histones that are commonly referred to as epigenetic marks. Such alterations (i.e., methylation, acetylation, and ubiquitination) are catalyzed by an array of dedicated enzymes with antagonistic activity, including methyltransferases and demethylases, acetyltransferases and deacetylases, as well as ubiquitin ligases and deubiquitinating enzymes. The epigenetic control of transcription is critical not only for embryonic and postembryonic development but also for the preservation of homeostasis in all adult tissues. In line with this notion, epigenetic defects have been associated with a variety of human disorders, including (but not limited to) congenital conditions as well as multiple hematological and solid tumors. Here, we provide an in-depth discussion of the impact of epigenetic alterations on cancer stemness, i.e., the ability of a small population of poorly differentiated malignant cells to (1) self-renew while generating a more differentiated progeny, and (2) exhibit superior tumor initiating/repopulating potential along with exceptional plasticity and improved resistance to environmental and therapy-elicited stress. Moreover, we critically evaluate the potential and limitations of targeting epigenetic modifiers as a means to eradicate cancer stem cells for therapeutic purposes.

## Introduction

**Fig. 1:**
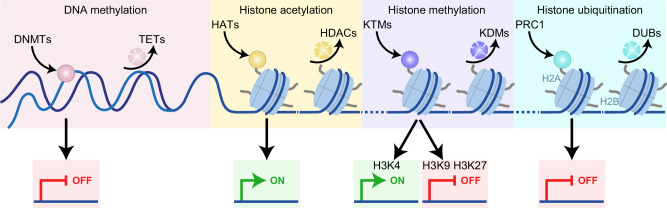
Major epigenetic mechanisms of transcriptional regulation. Multiple epigenetic modifications regulate transcription. DNA methylation, which is catalyzed by DNA methyltransferases (DNMTs) and reversed by Tet methylcytosine dioxygenases (TETs), typically represses transcription by impairing transcription factor (TF) binding. In contrast, histone posttranslational modifications (PTMs), including acetylation, methylation, and ubiquitination, modulate chromatin structure and transcriptional accessibility. Histone acetylation is regulated by histone acetyltransferases (HATs) and histone deacetylases (HDACs), and promotes gene expression by opening chromatin. In contrast, the methylation of specific histone residues, which is regulated by lysine histone methylases (KTMs) and lysine demethylases (KDMs), has functional consequences that are influenced by the position of the residue and the degree of methylation. Similarly, histone ubiquitination, which is catalyzed by E1-activating-E2 conjugating-E3 ligase systems, including polycomb repressive complex 1 (PRC1), and is reversed by deubiquitinating enzymes (DUBs), with histone 2A (H2A) and H2B as the main targets, regulates gene expression in a context-dependent manner. Notably, these marks not only affect local promoter activity but also regulate distal elements such as enhancers. Moreover, epigenetic modifications often act in concert, with extensive crosstalk between DNA methylation and histone PTMs, either synergistically or antagonistically influencing gene expression, partly through the recruitment of reader proteins and chromatin remodeling complexes

Genetic information is decoded to enable cellular functions via a finely regulated multistep process that initiates with DNA-to-RNA transcription.^1,2^ Thus, the expression of specific transcription factors (TFs) and their binding partners is fundamental for a cell to acquire precise biological features, in both physiological and pathological settings, such as cancer.^3^ Importantly, the conversion of genetic information into cellular functions, as initiated by transcription, is controlled by a number of pretranscriptional mechanisms, notably by modifications of DNA, histones, and chromatin structure.^4^ These modifications, which are commonly known as epigenetic marks, are reversible but can be transmitted across generations, thereby preserving the memory of gene activity while enabling transcriptomic plasticity in response to developmental and environmental cues (Fig. 1).^5^

Epigenetic modifications are critical for embryonic and postembryonic development as well as for the preservation of adult tissue homeostasis.^6,7^ Historically, the term epigenetics was first introduced by Conrad H. Waddington in 1942 to describe how the interaction between genotype and the environment shapes phenotypes during development, particularly through the regulation of cell fate and differentiation.^8^ Epigenetic traits specifically regulate the expression of pluripotency and cell lineage genes in a developmental stage-, organ- and cell type-specific manner.^9,10^ In line with this notion, defects in the epigenetic control of gene expression have been associated with a number of human disorders, including cancer.^11,12^ Accordingly, several drugs with prominent epigenetic effects, including azacitidine, decitabine and various histone deacetylase (HDAC) inhibitors, are currently licensed for use in cancer patients.^13,14^

Preclinical and clinical findings demonstrate that some tumor types (especially, but not exclusively, hematological malignancies) rely on a poorly differentiated population of neoplastic cells that can self-renew while generating more differentiated cellular progeny, and exhibit superior resistance to adverse microenvironmental conditions and immune elimination, which are commonly referred to as cancer stem cells (CSCs).^15–17^ Owing to these features, CSCs stand out as crucial drivers of oncogenesis, disease progression, and treatment resistance.

**Fig. 2:**
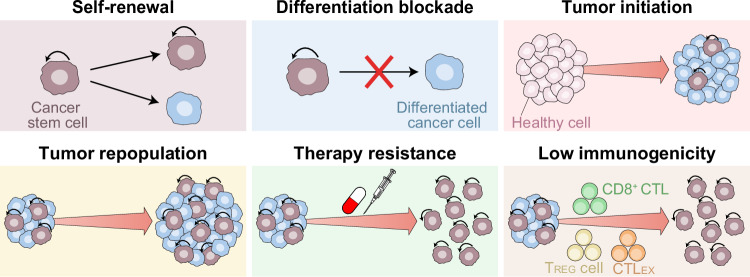
CSC features influenced by epigenetic processes. Epigenetic mechanisms contribute to the maintenance of cancer stem cell (CSC) identity by: (1) enabling an indefinite proliferative capacity; (2) preserving an undifferentiated cellular state; (3) promoting tumor initiation and repopulation by supporting asymmetric division, which allows CSCs to simultaneously self-renew and generate differentiated progeny, thereby replenishing the tumor mass; (4) increasing treatment resistance through mechanisms such as quiescence, enhanced DNA damage repair, efficient reactive oxygen species (ROS) detoxification, and limited apoptotic sensitivity; and (5) driving immune evasion mechanisms, including defective antigen presentation, immunosuppressive cytokine production, and the upregulation of coinhibitory immune checkpoints, which allow CSCs to escape immunosurveillance. CTL cytotoxic T lymphocyte, CTL_EX_ exhausted CTL, T_REG_ regulatory T cell

Emerging evidence suggests that not only genetic traits, but also highly plastic epigenetic mechanisms support key features of cancer stemness, including (1) their ability to self-renew in the context of arrested differentiation, (2) their superior tumor-initiating and repopulating potential, and (3) their pronounced capacity to resist stress and evade cancer immunosurveillance (Fig. 2).^17–19^ Importantly, epigenome profiling revealed that CSCs share epigenetic traits with embryonic stem cells, such as the repression of genes associated with cell differentiation, but not necessarily with adult stem cells,^20–23^ hence representing potential targets for the development of novel anticancer therapies.^24^

Here, we review the molecular mechanisms through which the epigenetic control of transcription by DNA and histone modifications governs key features of cancer stemness, including self-renewal, differentiation blockade, as well as tumor initiation and propagation. Moreover, we discuss the role of epigenetic regulators in cancer stemness-associated plasticity, as we critically evaluate therapeutic strategies targeting epigenetic modifiers to overcome CSC-driven resistance to treatment. Throughout the article, we focus on tumor types with a well-defined CSC-dependent hierarchical organization, including acute myeloid leukemia (AML), chronic myeloid leukemia (CML), glioblastoma (GBM), colorectal cancer (CRC), and breast cancer. Conversely, how posttranscriptional RNA modifications (e.g., epitranscriptomic changes) impact the biology of CSCs will not be discussed in this Review. Similarly, the impact of epigenetic alterations on the immunoevasive properties of CSCs has recently been reviewed in detail^18^ and hence will not be further discussed here.

## Epigenetic control of cancer stemness and its relevance for oncogenesis

**Fig. 3:**
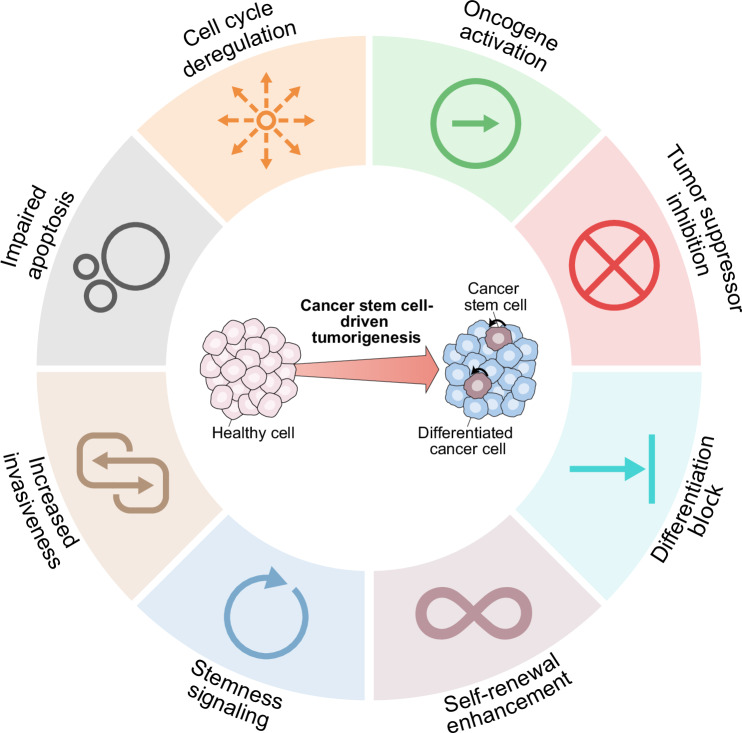
Epigenetic regulation of CSC-related oncogenesis and tumor progression. DNA methylation, histone methylation, histone acetylation, and histone ubiquitination regulate a number of genetic programs that sustain the tumor-forming and repopulating capacities of cancer stem cells (CSCs), including programs that: (1) enable and preserve self-renewal, (2) prevent cellular differentiation, (3) activate oncogenic signaling and/or inactivate cancer cell-intrinsic oncosuppression, (4) deregulate cell cycle control and apoptotic cell death, and (5) promote local invasiveness and metastatic dissemination. This figure summarizes findings from tumors with a recognized CSC-driven cellular hierarchy, including acute and chronic myeloid leukemia, glioblastoma, colorectal carcinoma, and breast cancer

Epigenetic mechanisms support cancer stemness by enabling and preserving long-term self-renewal while suppressing cellular differentiation. This is mediated by the deregulated expression of pluripotency factors such as POU class 5 homeobox 1 (POU5F1, also known as OCT4), SRY-box transcription factor 2 (SOX2), and Nanog homeobox (NANOG), along with (1) the aberrant activation of stemness-related pathways, including WNT, NOTCH, and Hedgehog signaling, and (2) the repression of lineage-specifying transcriptional programs, such as those governed by HOX gene clusters.^25^ Together, these alterations disrupt the balance between stem-like identity and lineage commitment. Epigenetic changes can trigger additional genetic programs that reinforce CSC properties and sustain their tumor-forming and tumor-repopulating capacities, including programs leading to the activation of oncogenic signaling, the silencing of tumor suppressor pathways, the deregulation of cell cycle and apoptosis, and the promotion of invasion and metastasis (Fig. 3), as outlined below.

### DNA methylation

CSCs exhibit distinct epigenetic landscapes compared with bulk tumor cells, differentiated cancer cells, as well as normal stem cells, with (1) a prevalence of epigenetic signatures associated with accelerated cellular proliferation and disease pathogenesis, and (2) signs of deregulated activity of DNA-methylating and demethylating enzymes, which are globally linked to CSC preservation.^26,27^ For example, while DNA methyltransferase 1 (DNMT1) is crucial for maintaining normal and malignant stem cells by sustaining DNA methylation patterns in support of self-renewal,^28,29^ only CSCs require DNMT1 expression for survival, indicating a unique role of this enzyme only in the latter.^29^ Accordingly, DNMT1 has been shown to promote cancer stemness and tumorigenicity in multiple hematological and solid malignancies by sustaining pluripotency and stemness-related programs while suppressing differentiation pathways.^29–32^ In AML, DNMT1 promotes leukemogenesis by repressing tumor suppressor and differentiation genes through a mechanism involving DNA hypermethylation and the establishment of bivalent chromatin marks mediated by enhancer of zeste 2 polycomb repressive complex 2 subunit (EZH2, see below).^29^ In breast cancer, DNMT1 promotes CSC-driven oncogenesis by hypermethylating and silencing TFs that balance stemness and differentiation, such as ISL LIM homeobox 1 (ISL1)^28^ and forkhead box O3 (FOXO3).^30^ This repression can lead to the upregulation of pluripotency-associated genes. For example, *FOXO3* hypermethylation results in the expression of SOX2, which enhances self-renewal and transactivates DNMT1 in a feed-forward loop.^30^ In CRC, DNMT1 contributes to CSC maintenance by silencing genes involved in differentiation and apoptosis.^31,33^ Dysregulated DNA methylation can also promote cancer stemness by activating WNT/β-catenin signaling. For example, in hepatocellular carcinoma (HCC), the DNMT1-regulated protein brain-expressed X-linked 1 (BEX1) is overexpressed and sustains CSC maintenance by sequestering RUNX family transcription factor 3 (RUNX3), a repressor of catenin beta 1 (CTNNB1) transcription, thereby activating WNT/β-catenin signaling.^34^ Aberrant DNA methylation also disrupts intestinal stem cell (ISC) differentiation during early WNT/β-catenin-driven tumorigenesis.^35^

Alterations in the DNA methylation‒demethylation balance that support cancer stemness can also result from dysregulated tet-methylcytosine dioxygenase 2 (TET2) activity. In GBM, SOX2 contributes to the preservation of self-renewal and enhances the tumor-propagating potential of glioma stem cells (GSCs) via a mechanism involving the indirect inhibition of TET2.^36^ Consistently, TET2 reconstitution suppresses tumor growth and improves survival in orthotopic GBM models.^36^ In this context, circulating tumor cells, which share increased tumor-repopulating potential with CSCs, exhibit hypomethylation at several CSC-related genetic loci, including *SOX2*, *POU5F1,* and *NANOG*,^37^ indicating the existence of strong epigenetic regulation of stemness-related TFs.

A similar pattern of TET2 dysregulation has been observed in hematological malignancies, particularly in AML, a setting in which *TET2* mutations are frequent^38^ and contribute to leukemia stem cell (LSC) generation, expansion, and maintenance. Mechanistically, TET2 loss induces hypermethylation and repression of genes involved in hematopoietic differentiation, such as GATA binding protein 2 (GATA2) and members of the HOX gene family,^39–42^ thereby reinforcing self-renewal and stemness potential. Among these genes, *GATA2* appears to play a particularly critical role in leukemogenesis driven by TET2 loss.^40^ Confirming its ability to suppress AML, restoring TET2 expression prevents leukemogenesis.^43,44^ Moreover, branched chain amino acid transaminase 1 (BCAT1) activity has been shown to support the in vivo engraftment capacity of LSCs by altering the epigenomic landscape toward widespread hypermethylation via disrupted α-ketoglutarate homeostasis, which is a key endogenous inhibitor of TET enzymes.^45^ Similarly, mutations in isocitrate dehydrogenase (NADP(+)) 1 (*IDH1*) and *IDH2*, which are common in GBM and hematological tumors,^46^ lead to the synthesis of the oncometabolite *D*-2-hydroxyglutarate, which inhibits TET enzymes and causes widespread DNA hypermethylation, supporting the maintenance of LSCs while limiting differentiation.^47^

Additional mechanisms linking increased DNA methylation to CSC maintenance and tumorigenicity include: (1) the activation of cellular quiescence, as mediated by DNMT1 through its interaction with the CSC marker prominin 1 (PROM1, best known as CD133), and the upregulation of the cell cycle inhibitors cyclin-dependent kinase inhibitor 1A (CDKN1A, best known as p21) and CDKN1B (best known as p27) in GSCs,^32^ and (2) the increased migration and homing of LSCs to bone marrow niches, as mediated by TET2 deficiency through the upregulation of tetraspanin 13 (TSPAN13) and the activation of CXCR4 signaling, which promotes LSC proliferation and self-renewal.^44^

However, self-renewal can also be suppressed by active DNA hypermethylation and sustained by DNA demethylation. For example, *IDH1* mutations coupled with *D*-2-hydroxyglutarate accumulation suppress stemness in GBM downstream of inhibited WNT/β-catenin signaling,^48^ whereas BMP signaling restricts the GSC compartment by favoring the DNMT3A-mediated methylation of *PROM1*.^49^ Moreover, TET1 enhances the expression of *NANOG* and other pluripotency genes in brain neoplasms by increasing 5-hydroxymethylcytosine (5hmC) marks.^50,51^ Notably, TET1 has both tumor suppressive^52^ and oncogenic^53,54^ effects. The latter involves transactivation of leukemogenic genes such as homeobox A9 (*HOXA9*) and Meis homeobox 1 (*MEIS1*) upon interaction with chimeras involving lysine methyltransferase 2A (KMT2A, also known as MLL), or activation of transcriptional programs dependent on signal transducer and activator of transcription 5B (STAT5B).^53,54^ Similarly, TET2 liquid-like condensation with KMT2B and lysine demethylase 6A (KDM6A) has been recently identified as a crucial mechanism for accurate DNA demethylation, and disruption of this complex results in widespread DNA demethylation errors coupled with impaired leukemia progression.^42^ Finally, mutations in *DNMT3A*, which are frequent in AML and other hematologic cancers,^55^ have been associated with increased LSC self-renewal, possibly due to blocked cell differentiation.^56,57^ Accordingly, these mutations contribute to AML development via the hypomethylation-mediated transactivation of *MEIS1*, which encodes a transcriptional cofactor that activates leukemogenic gene programs, enforcing self-renewal and blocking differentiation.^58^

**Fig. 4:**
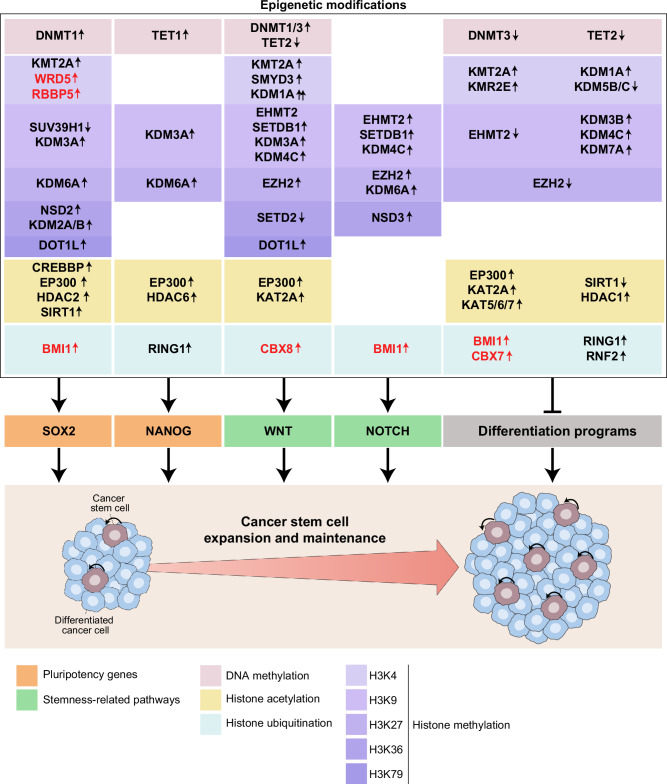
Epigenetic regulation of CSC self-renewal. DNA methylation, histone methylation, histone acetylation, and histone ubiquitination are implicated in the control of genetic programs regulating the preservation of self-renewal in cancer stem cells (CSCs). These programs involve not only the epigenetic activation of pluripotency factors such as SOX2 and NANOG but also (1) the activation of signal transduction cascades that support stemness, such as WNT/β-catenin and NOTCH signaling, and (2) the repression of gene sets promoting cellular differentiation. This figure summarizes findings from tumors with a recognized CSC-driven cellular hierarchy, including acute and chronic myeloid leukemia, glioblastoma, colorectal carcinoma, and breast cancer. ↑, increased activity or expression; ↓, decreased activity or expression. Proteins listed in red lack recognized catalytic activity but regulate the functions of bona fide epigenetic modifiers (black)

Collectively, these observations demonstrate that aberrant DNA methylation patterns influence cancer stemness mostly by controlling pluripotency and stemness-related programs while suppressing differentiation (Fig. 4). However, the precise impact of these epigenetic alterations is highly context dependent, varying across different cancer types and depending on the specific genomic loci affected.

### H3K4 methylation

CSC maintenance is highly dependent on the activity of enzymes regulating H3K4 methylation, a histone mark typically associated with active transcription.^5^ Methyltransferases of the KMT2 and SMYD families have indeed been reported to sustain stemness in leukemia and multiple solid tumors by transactivating stemness-related genes upon the deposition of H3K4me3 marks.^59–62^ For example, KMT2A regulates pancreatic CSC self-renewal by promoting the expression of pluripotency TFs, including SOX2, upon association with an RNA polymerase-associated subcomplex.^59^ It also sustains colorectal CSC self-renewal and tumor-initiating capacity by favoring the transactivation of WNT/β-catenin genes, including leucine-rich repeat-containing G protein-coupled receptor 5 (*LGR5*, an intestinal and colorectal stem marker),^60^ a mechanism that may also be relevant for preserving ISCs.^60,63^ In line with these findings, KMT2A depletion reduces in vivo tumorigenicity by inducing p21-dependent cell cycle arrest in the absence of overt apoptosis.^60^ Similarly, the histone methyltransferase SET and MYND domain containing 3 (SMYD3) favors colorectal CSC maintenance by participating in a feedback loop involving deregulated WNT/β-catenin signaling.^62^

Components of the so-called WRAD complex, a supramolecular entity enabling optimal histone methylation by KMT2s,^64^ such as WD repeat domain 5 (WDR5) and RB binding protein 5, histone lysine methyltransferase complex subunit (RBBP5), play roles in GSC preservation by stimulating *SOX2* and *POU5F1* expression.^65,66^ WDR5 inhibition disrupts tumor propagation in GBM models,^65^ indicating a key role for WDR5-regulated histone marks in GSC-driven oncogenesis. Similarly, dpy-30 histone methyltransferase complex regulatory subunit (DPY30, another WRAD complex component) promotes the tumorigenic potential of GSCs by epigenetically activating transcriptional programs linked to angiogenesis and hypoxia responses.^67^

Notably, the regulation of CSC self-renewal often relies on the interplay between multiple epigenetic modifiers. For example, in MLL-AF4 leukemias, KMT2A overexpression promotes CSC self-renewal and propagation by transactivating *PROM1* through functional collaboration with the H3K79 methylase DOT1-like histone lysine methyltransferase (DOT1L) (see below).^61^ Moreover, ALF transcription elongation factor 1 (AFF1, best known as AF4), which is part of the MLL-AF4 fusion in some pediatric acute lymphoblastic leukemias, was shown to directly regulate *PROM1* transcription in concert with DOT1L, and its loss impairs leukemogenesis.^68^ KMT2A and KMT2E also preserve self-renewal and limitless proliferative potential in CSCs by counteracting the differentiating effects of the histone variants macroH2A2 and H3.3, potentially through alterations in general chromatin organization.^69,70^ In this context, KMT2E enhances GSC tumorigenicity by repressing neural differentiation programs such as interferon and NEUROD4 signaling.^70^ Conversely, KMT2C, which is frequently mutated in HCC, non-small cell lung carcinoma (NSCLC), and breast cancer,^71^ appears to limit CSC-driven tumorigenicity, at least in breast cancer, as its loss is linked to the acquisition of undifferentiated features in the context of the epithelial‒mesenchymal transition (EMT).^72^

Further confirming the role of H3K4 methylation dynamics in cancer stemness, KDM1A (also known as LSD1) has been shown to support CSC function and leukemogenesis downstream of MLL-AF9 translocations by impairing LSC differentiation and apoptosis.^73^ KDM1A also appears to promote leukemogenesis by (1) enhancing HSC self-renewal upon activation of the MEIS1-HOXA axis^74^ and (2) interacting with EMT-promoting factors such as snail family transcriptional repressor 1 (SNAI1), redirecting SNAI1 activity from HSC regulation to repression of cell adhesion and oncosuppressor genes.^75^

KDM1A has also been suggested to promote the oncogenic potential of GSCs by repressing the transcription of genes involved in cell cycle inhibition, differentiation, and apoptosis, including bone morphogenetic protein 2 (*BMP2*) and *CDKN1A*.^76,77^ Accordingly, KDM1A inhibition reduces tumor progression and extends survival in preclinical GBM models.^76,78^ Moreover, KDM1A enhances WNT/β-catenin signaling in HCC and thyroid cancer by suppressing the transcription of genes encoding WNT antagonists such as APC regulator of WNT signaling pathway (APC), and dickkopf WNT signaling pathway inhibitor 1 (DKK1), ultimately promoting stemness.^79,80^ In this context, the WNT/β-catenin pathway regulator glycogen synthase kinase 3 beta (GSK3B) promotes KDM1A stabilization through direct phosphorylation, resulting in inhibited differentiation and enhanced GSC self-renewal.^77^ KDM1A has also been reported to epigenetically activate and interact with BMI1 proto-oncogene, polycomb ring finger (BMI1), a master regulator of self-renewal (see below).^81^ In contrast to KDM1A, KDM5 family members appear to restrict cancer stemness, at least in AML. Specifically, KDM5C limits the expression of dedifferentiation genes, maintaining them in a bivalent repressed state,^82^ whereas KDM5B represses stemness-related genes through chromatin binding, independent of its histone demethylation activity.^83^

Taken together, these observations point to the complex role of H3K4 methylation dynamics in cancer stemness and CSC-driven oncogenesis across tissue types, with both methyltransferases (KMT2A) and demethylases (KDM1A) contributing to CSC maintenance by promoting self-renewal while limiting cellular differentiation (Fig. 4).

### H3K9 methylation

CSCs exhibit defects in multiple enzymes involved in H3K9 methylation, a histone mark that is typically linked to gene repression.^5^ H3K9 methyltransferases contribute to the epigenetic regulation of pluripotency, stemness-associated genes, and differentiation programs, often mediating context-specific effects on self-renewal, CSC preservation, and tumorigenicity. For example, SUV39H1 histone lysine methyltransferase (SUV39H1) inhibits stemness in melanoma and AML upon the deposition of H3K9 marks.^84,85^ In melanoma, H3K9 methylation as mediated by SUV39H1 or euchromatic histone lysine methyltransferase 1 (EHMT2, also known as G9a), results in SOX2 downregulation, thereby restricting CSC self-renewal.^85^ In MLL-rearranged AML, SUV39H1 suppresses LSC self-renewal and leukemogenesis by downregulating TFs involved in LSC maintenance, such as HOXB13, SIX homeobox 1 (SIX1), MEIS1, and HOXA9.^84^ In contrast, EHMT2 reportedly promotes LSC-driven AML development by interacting with HOXA9 at HOXA9-dependent transcriptional sites, *de facto* boosting leukemogenic transcriptional programs while supporting LSC proliferation and self-renewal.^86^ In CML, EHMT2 drives leukemogenesis by repressing oncosuppressors such as the TF SOX6, and pharmacological EHMT2 inhibitors effectively eradicate LSCs and prolong survival in mouse CML models.^87^

Deregulated H3K9 methylation can also lead to the activation of stemness-related pathways. EHMT2 activity plays a crucial role in maintaining stemness in CRC^88^ and melanoma.^89^ More specifically, while in melanoma, EHMT2 silences *DKK1*, leading to the derepression of WNT/β-catenin signaling,^89^ in CRC, it promotes epigenetic reprogramming to promote oncogenesis through WNT/β-catenin signaling activation and EMT induction, as demonstrated by pharmacological EHMT2 inhibitors.^88^

In support of the notion that multiple epigenetic regulators cooperate to maintain CSC identity, EHMT2 functionally cooperates with the polycomb repressive complex 2 (PRC2) via ligand-dependent nuclear receptor corepressor (LCOR) in prostate cancer, resulting in dual H3K9/K27 methylation and consequent repression of differentiation genes.^90^ Moreover, SET domain bifurcated histone lysine methyltransferase 1 (SETDB1), in combination with EZH2 (see below), promotes CSC self-renewal in skin cancer by silencing RUNX family transcription factor 3 (RUNX3),^91^ a TF that inhibits stemness by suppressing WNT/β-catenin and NOTCH signaling.

H3K9 demethylases generally promote CSC maintenance and tumorigenicity. For example, KDM3A enhances cancer stemness by epigenetically activating key genes such as *SOX2* and *NANOG*, at least in the context of ovarian cancer.^92^ Moreover, KDM3 family members and KDM4C support the tumorigenicity of colorectal CSCs^93^ and GSCs,^94^ respectively, via WNT/β-catenin signaling. Mechanistically, KDM3s epigenetically activates WNT/β-catenin signaling by removing repressive H3K9me2 marks and facilitating H3K4 methylation via KMT2A at WNT/β-catenin target genes.^93^ Similarly, KDM4C supports GSC self-renewal by enhancing WNT/β-catenin signaling through interaction with *CTNNB1* and epigenetic activation of transcription factor 4 (*TCF4*).^84^ KDM4C also sustains colorectal CSCs and promotes colorectal carcinogenesis upon activation of NOTCH signaling via AT-rich interaction domain 3B (ARID3B)-mediated chromatin recruitment.^95^ Moreover, both KDM4C and KDM7A promote stemness in GBM by repressing various genes involved in differentiation.^96^

Finally, pro-leukemogenic effects have been attributed to KDM3B and KDM4C. KDM3B activates HOXA9-controlled transcriptional programs that are critical for LSC (but not HSC) maintenance through physical interactions with HOXA9.^97^ Similarly, KDM4C enhances LSC self-renewal via transactivation of alkB homolog 5, RNA demethylase (ALKBH5), leading to increased expression of oncogenic factors such as AXL receptor tyrosine kinase (AXL) and transforming acidic coiled-coil containing protein 3 (TACC3).^98,99^

In summary, H3K9 demethylases (KDM3, KDM4, and KDM7 family members) preserve the CSC pool and functionality by activating stemness pathways while repressing differentiation, whereas H3K9 methyltransferases exhibit context-dependent effects, either sustaining or inhibiting CSC maintenance (Fig. 4).

### H3K27 methylation

LSCs present a unique H3K27 methylation profile generally due to the deregulation of EZH2,^100,101^ which exerts methyltransferase activity as a part of PRC2.^102^ EZH2 exhibits recurrent gain-of-function mutations (e.g., in germinal center B-cell lymphomas) and is overexpressed in several solid tumors, thus sustaining stemness and promoting oncogenesis across both hematological and solid malignancies.^103–105^ In GSCs, EZH2 supports both maintenance and tumorigenicity by transactivating MYC proto-oncogene, bHLH transcription factor (*MYC*, also known as c-MYC), an oncogene involved in pluripotency.^106^ It also promotes CSC-mediated tumorigenicity in breast cancer by enhancing WNT/β-catenin signaling.^107^

In line with a role for EZH2 in the transcriptional repression of differentiation-associated genes, EZH2 preserves stemness in CRC by maintaining the promoter of the indian hedgehog signaling molecule (*IHH*), which encodes a colonocyte differentiation factor, in a bivalent repressive state.^103^ In GSCs, EZH2 also enhances STAT3 signaling through a mechanism unrelated to its canonical epigenetic functions but rather involves direct methylation and binding of STAT3.^105^ Moreover, EZH2 indirectly preserves LSC self-renewal by maintaining them in a quiescent state via the repression of cyclin D1 (*CCND*) transcription.^104^ In CRC, EZH2 repression also contributes to tumor evolution. Indeed, recent findings delineate a mechanism of colorectal carcinogenesis in which APC-deficient, telomere-dysfunctional CSCs outcompete adjacent ISCs by promoting their differentiation, at least in part reflecting EZH2 repression driven by telomere dysfunction coupled with the secretion of WNT antagonists with paracrine activity.^108^

H3K27 methylation by EZH2 also contributes to leukemogenesis. In CML, EZH2 inactivation leads to contraction of the LSC compartment and impaired disease maintenance in vivo, independent of BCR-ABL1 status.^100^ In AML, EZH2 facilitates tumorigenesis by repressing phosphatase and tensin homolog (PTEN), which encodes a major tumor suppressor.^101^ Notably, dual deletion of EZH1 and EZH2 from AML cells results in complete remission through increased differentiation, suggesting a critical function of these epigenetic modifiers in leukemogenesis.^104^ However, whether such a function is mechanistically linked to the LSC compartment remains to be determined.

The impact of H3K27 demethylases on CSC self-renewal is context-dependent. For example, KDM6A appears to support stemness in solid tumors responding to therapy by: (1) activating pluripotency genes,^109^ (2) cooperating with H3 acetylation as mediated by E1A binding protein p300 (EP300), at least in the context of breast cancer,^110^ or (3) promoting a switch toward reduced proliferation coupled with a global redistribution of H3K27 marks and upregulated NOTCH signaling, as observed in GSCs.^111^ However, mutations in *KDM6A* are common in multiple cancer types, potentially suggesting a suppressive role in CSC maintenance.

Thus, while the H3K27 methyltransferase EZH2 consistently supports cancer stemness and CSC-mediated oncogenesis, the role of H3K27 demethylases such as KDM6A is highly context dependent (Fig. 4).

### Other histone methylations

H3K36 methyltransferases can either support or suppress self-renewal, at least in some settings, through mechanisms that are also active in normal stem cells.^112^ SET domain containing 2, histone lysine methyltransferase (*SETD2*) is frequently mutated in multiple neoplasms, including breast and lung cancers, and contributes to cancer progression through increased stemness and dysregulation of differentiation pathways. SETD2 mutations drive leukemogenesis by impairing normal hematopoiesis while supporting LSC self-renewal and tumorigenicity. This is mediated by decreased H3K36me3 marks, which alter differentiation- and cell cycle-regulatory programs, such as KLF1-related programs,^113^ and activate stemness-associated pathways, such as WNT/β-catenin signaling.^114^ In contrast, ASH1-like histone lysine methyltransferase (ASH1L) facilitates leukemogenesis in the context of increased stemness by recruiting PC4 and SRSF1 interacting protein 1 (PSIP1, best known as LEDGF) and MLL-containing chimeras to transcriptionally activate leukemia-relevant genes, an effect antagonized by the H3K36 demethylase KDM2A.^115^ Similarly, nuclear receptor binding SET domain protein 2 (NSD2) drives self-renewal by transactivating *SOX2* and *POU5F1* in distinct solid tumors,^116,117^ although a role in promoting CSC immunosurveillance has also been reported.^115^ Moreover, NSD3 sustains CSC self-renewal in breast cancer by stimulating H3K36me2/3-dependent activation of NOTCH signaling.^118^ Notably, both NSD2 and NSD3 are frequently overexpressed in multiple oncological settings. Among H3K36 demethylases, KDM2A epigenetically activates pluripotency genes in HCC,^119^ whereas KDM2B upregulation reportedly maintains LSCs and GSCs.^120,121^ In GBM, KDM2B depletion indeed leads to a decreased CSC compartment along with reduced levels of SOX2 and EZH2.^120^ Moreover, KDM2B exerts pro-leukemogenic effects by promoting MEIS1-HOXA9 signaling and LSC self-renewal through the repression of CDKN2B (best known as p15) via H3K36me2 demethylation.^122^

There is evidence for a role for the H3K79me2 methyltransferase DOT1L in the preservation of CSC self-renewal. DOT1L activity is indeed increased in LSCs compared with normal HSCs, reshaping the H3K79me2 landscape.^121^ DOT1L supports leukemogenesis driven by MLL-AF9 chimeras by modulating the accessibility of its targets.^123^ Accordingly, DOT1L loss significantly reduces LSC tumorigenicity, impairing leukemia maintenance in vivo.^123^ In this context, histone acetylation at the *DOT1L* locus by CREB binding protein (CREBBP) stabilizes DOT1L expression, further enhancing leukemogenesis.^123^ DOT1L also cooperates with MLL-AF4 to transactivate stemness-related genes such as *PROM1*.^61^ It also maintains stemness by promoting the expression of SOX2 and oligodendrocyte transcription factor 2 (OLIG2) in GSCs^124^ and by driving WNT/β-catenin signaling in gastric and breast cancer.^125–127^ At least in the latter setting, this also involves H3 acetylation by EP300 as well as MYC activation.^127^

Histone arginine methyltransferases also play critical roles in CSC biology in both solid and hematological cancers.^128^ Among them, protein arginine methyltransferase 5 (PRMT5) is implicated in breast CSC maintenance by controlling the expression of stemness-related TFs such as POU5F1, MYC, KLF transcription factor 4 (KLF4), and forkhead box P1 (FOXP1).^129,130^ In leukemia, PRMT5 supports LSC self-renewal, survival, and tumorigenicity by promoting WNT/β-catenin signaling (an action shared with PRMT1),^131^ as well as by repressing the tumor protein p53 (TP53, best known as p53) system.^132^ Specifically, PRMT5 directly methylates p53 at arginine residues, leading to the selective repression of p53-regulated oncosuppressive target genes. Other PRMT family members, such as PRMT6, PRMT7, and PRMT9, resemble PRMT5 in their ability to increase LSC survival and tumorigenicity.^133–135^ However, PRMT1 has also been shown to suppress leukemogenesis upon interaction with the cell cycle inhibitor BTG anti-proliferation factor 2 (BTG2),^136^ potentially indicating a context-dependent role for PRMTs in LSC biology.

PRMT2, PRMT3, and PRMT6 are also involved in GSC maintenance,^137–139^ although in the case of PRMT6, this may not be mediated by histone methylation. Moreover, PRMT6, PRMT7, and PRMT9 have been suggested to support LSC emergence through epigenetic alterations that directly influence cellular metabolism.^134,135,137^

In summary, H3K36 demethylases, the H3K79 methyltransferase DOT1L, and histone arginine methyltransferase play critical roles in supporting CSC self-renewal and tumorigenicity in various neoplasms, whereas the role of H3K36 methyltransferases in stemness remains context dependent (Fig. 4).

### Histone acetylation

Histone acetyltransferases (HATs) support CSC self-renewal by increasing the expression of stemness-related genes through the acetylation of lysine residues on histone tails at gene enhancers and promoters. CREBBP and EP300, two HATs with identified mutations in lymphomas and other malignancies, appear to be prominent modulators of this effect. Thus, CREBBP drives the emergence and expansion of GSCs via the epigenetic activation of stabilin 2 (*STAB2*), forkhead box M1 (*FOXM1*), and other stemness-associated genes.^140^ In this context, FOXM1 activation by CREBBP is essential for sustaining GSC tumorigenicity.^140^ A similar role for FOXM1 has been reported in uveal melanoma, a setting in which EP300 upregulates *ALKBH5* via H3K27 acetylation, resulting in FOXM1 overexpression upon *FOXM1* mRNA m⁶A demethylation and consequent activation of the EMT.^141^ In breast CSCs, EP300 cooperates with KDM6A to transcriptionally activate various pluripotency genes.^110^

HATs also support cancer stemness by enhancing WNT/β-catenin signaling, as demonstrated by EP300, which contributes to CRC stemness and tumorigenicity at least in part by transactivating *LGR5* through a mechanism that relies on jade family PHD finger 3 (JADE3).^142^ EP300 also cooperates with DOT1L to promote the epigenetic derepression of cadherin 1 (*CDH1*) in breast cancer,^127^ whereas KAT2A maintains pancreatic CSCs by acetylating H3 on various promoters and enhancers that control WNT/β-catenin signaling.^143^

Multiple HATs, including KAT2A,^144^ EP300,^145^ KAT7,^146^ KAT5,^147^ and KAT6,^148^ reshape the transcriptional LSC landscape to limit cellular differentiation, maintain stemness, and promote tumorigenicity. For example, KAT2A activity sustains transcriptional networks supporting LSC survival and tumorigenicity in AML without impacting HSCs.^144^ KAT2A also promotes AML development by increasing the expression of MYC and modulating its transcriptional functions.^149^ Accordingly, KAT2A loss drives shrinkage in the LSC compartment by limiting self-renewal while promoting differentiation and inducing apoptosis, impairing AML progression overall.^144,149^

EP300 facilitates leukemogenesis by promoting the exit of HSCs from quiescence and their malignant transformation through the acetylation of genes from the HOX family.^145^ It also interacts with chromatin accessibility regulators, such as high mobility group nucleosome binding domain 1 (HMGN1), to limit LSC differentiation and support self-renewal.^150^ Similarly, KAT7 promotes LSC-driven leukemogenesis in AML by modulating transcriptional programs that maintain stemness.^146^ Moreover, increased KAT5 activity reportedly facilitates leukemogenesis driven by ZMYND11-MBTD1 chimeras through transactivation of oncogenes such as *HOXA*, *MEIS1*, MYB proto-oncogene, transcription factor (*MYB*), *MYC*, and *SOX4*, resulting in increased LSC self-renewal.^147^ KAT6A also sustains AML by initiating a transcriptional module in which H3K9 acetylation is recognized by MLLT1 super elongation complex subunit (MLLT1, best known as ENL), promoting transcriptional elongation in LSCs in the context of leukemogenic programs.^148^ By disrupting this signaling module, KAT6A inhibition indeed promotes LSC differentiation and exerts potent antileukemic effects in vivo.^148^

HDACs exhibit complex and often context-dependent effects on cancer stemness. In GBM, several HDACs converge to preserve the GSC compartment. Specifically, HDAC1 is not only upregulated by NANOG^151^ but also reinforces the tumorigenic potential of GSCs by repressing p53-mediated tumor suppression.^152^ HDAC2 enhances stemness and GSC-driven tumorigenesis in conjunction with transforming growth factor beta (TGF-β) signal transducers such as SMAD family member 3 (SMAD3) and SKI proto-oncogene (SKI), at least in part by upregulating genes such as *SOX2* and *OLIG2*.^153^ Moreover, HDAC3 fosters GSC-related gliomagenesis by engaging GLI1 signaling,^154^ whereas HDAC6 supports GSC maintenance by modulating Hedgehog signaling.^155^

HDACs are involved in the upregulation of pluripotency genes in other solid tumors. For example, HDAC6 reinforces the colorectal CSC compartment by transactivating *NANOG* through a mechanism that depends on interleukin 6 (IL6) and STAT3,^156^ whereas sirtuin 1 (SIRT1) is overexpressed in hepatic CSCs, a setting in which it epigenetically regulates *SOX2* in concert with DNMTs.^157^ As an extra layer of complexity, HDACs have been reported to sustain cancer stemness by regulating energy metabolism.^158^ Finally, under certain circumstances, some HDACs can limit cancer stemness by acting as oncosuppressors, as exemplified by SIRT1, which preserves the HSC pool and limits leukemogenesis by increasing TET2 activity^159^ and modulating the expression of key developmental genes.^160^

In summary, the dynamic regulation of cancer stemness involves complex and context-dependent interactions between HATs and HDACs (Fig. 4).

### Histone ubiquitination

The PRC1 component BMI1 plays a crucial role in preserving stemness in both normal and malignant tissues upon gene silencing via histone H2A monoubiquitination.^161,162^ However, experimental evidence supports the aberrant activity and unique role of BMI1 in CSCs. Thus, BMI1 acts as a major regulator of CSC self-renewal across multiple hematological and solid tumors, including AML, CRC, and brain cancer.^163–165^ In these malignancies, deregulated BMI1 expression and PRC1 activity enable transcriptional programs that sustain stem-like properties, thereby promoting cancer development and progression.

From a mechanistic perspective, PRC1 epigenetically activates stemness-related pathways to establish and maintain CSCs, as observed in solid tumors. More specifically, BMI1 upregulation has been shown to promote the conversion of normal mammary stem cells into CSCs upon the activation of Hedgehog signaling.^162^ In breast cancer, a similar activity has been ascribed to chromobox 8 (CBX8).^166^ In this context, CBX8 supports stemness and enhances tumorigenicity by triggering NOTCH signaling through the association of PRC1 with WDR5, which increases H3K4 trimethylation at the promoters of NOTCH-related genes.^166^ PRC1 activity also limits CSC differentiation, as shown for BMI1, which inhibits the expression of differentiation genes in GSCs, *de facto* preserving stemness.^167^ Accordingly, BMI1 inhibition induces cellular senescence in GSCs, resulting in suppressed oncogenesis.^167^ However, BMI1 and EZH2 appear to exhibit complementary functions in GBM tumorigenesis, supporting the survival and tumor-initiating abilities of mesenchymal and proneural GSCs, respectively.^164^

PRC1 is also emerging as a key regulator of the tumorigenic potential of LSCs. Both BMI1 and CBX promote the malignant conversion of normal HSCs into LSCs by enhancing self-renewal^163,168^ as well as preventing cellular differentiation and apoptosis.^169^ Moreover, various CBX family members facilitate leukemogenesis by promoting stemness through noncanonical interactions. For example, CBX8 interacts with MLL-AF9 and KAT5 to drive the expression of HOX family genes,^170^ whereas CBX7 interacts with H3K9 methyltransferases to inhibit differentiation and increase self-renewal.^171^ Notably, other PRC1 components, such as RING1 and ring finger protein 2 (RNF2), also sustain the maintenance and tumorigenic potential of LSCs by inhibiting the expression of the TF GLIS family zinc finger 2 (GLIS2), thereby repressing differentiation-associated genetic programs^172^ or tumor suppressors such as CDKN2A (best known as p16).^163,169^

These findings highlight the emerging role of PRC1, particularly its core component BMI1, as a key regulator of CSC self-renewal, differentiation, and tumorigenicity across multiple malignancies (Fig. 4).

## Epigenetic plasticity and cancer stemness

**Fig. 5:**
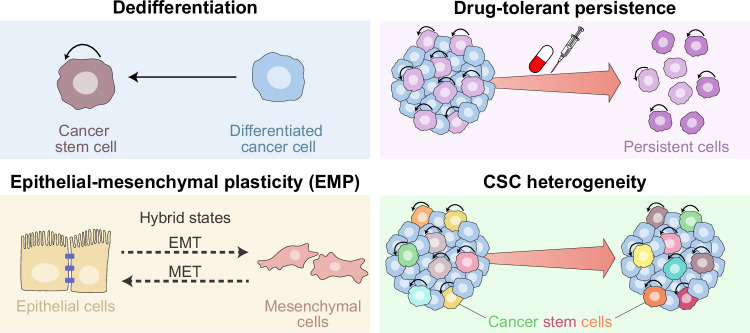
Epigenetic plasticity as a driver of cancer stemness. A number of plasticity programs sustain cancer stem cell (CSC) properties and tumor evolution through epigenetic mechanisms. These programs promote: (1) dedifferentiation to a stem-like state, whereby differentiated cancer cells revert to CSCs in response to oncogenic or environmental cues, including therapy, via chromatin remodeling and activation of pluripotency-associated transcriptional networks; (2) drug-tolerant persistence, in which a subpopulation of cancer cells survives therapy by entering a slow-cycling, stem-like state, forming a reservoir that seeds for relapse and resistance; (3) epithelial‒mesenchymal plasticity (EMP), referring to reversible transitions across hybrid epithelial‒mesenchymal transition (EMT) and mesenchymal‒epithelial transition (MET) states associated with stem‒like traits and increased metastatic potential; and (4) CSC heterogeneity, referring to the dynamic transitions across CSC states, contributing to tumor heterogeneity and adaptation

Cellular plasticity refers to the ability of a cell to undergo dynamic and reversible changes in identity, function, or phenotype in response to intrinsic or extrinsic cues.^173^ This process is tightly controlled by epigenetic mechanisms and occurs physiologically to support development, regeneration, and tissue homeostasis.^174^ However, (pre)malignant cells can hijack plasticity programs in support of malignant transformation, disease initiation, progression, and resistance to therapy.^175^ In particular, high plasticity enables (pre)malignant cells to escape fixed lineage constraints and adapt to changing microenvironmental conditions by transitioning between differentiated and stem-like states.^176^ In this section, we focus specifically on plasticity programs associated with the acquisition of CSC traits and the promotion of CSC heterogeneity (Fig. 5).

### Dedifferentiation to stem-like states during tumorigenesis and in response to therapy

Abundant evidence from cell lineage tracing, cell ablation, and single-cell sequencing studies has demonstrated that even fully differentiated cancer cells can revert to a stem-like state under specific conditions, including therapeutic pressure (Fig. 5). This process of vertical cell type transition is driven by epigenetic reprogramming and the activation of CSC-associated transcriptional programs enhancing survival, immune evasion, therapeutic resistance, and metastatic potential.^177–179^

For example, studies with patient-derived organoids, xenografts, and lineage tracing have shown high plasticity in CRC, where tumors can persist after CSC ablation through reacquisition of stem-like traits independent of niche signals.^180–182^ Similarly, in lung adenocarcinoma, oncogenic signaling as elicited by KRAS proto-oncogene, GTPase (*KRAS*) mutations initiate lineage reprogramming by activating ERK signaling.^183^ Extrinsic factors, including microenvironmental cues and therapeutic stress, can also drive cellular plasticity. In CRC, stem-like potential is acquired through local activation of WNT signaling induced by extrinsic signals, including (but not limited to) hepatocyte growth factor (HGF) released by cancer-associated fibroblasts (CAFs).^184–186^ In GBM, microenvironmental cues trigger epigenetically mediated cell-state transitions and the acquisition of stem-like traits.^187^ Moreover, treatment with temozolomide promotes the dedifferentiation of nonstem glioma cells into GSC-like cells via the upregulation of hypoxia inducible factor 1 subunit alpha (HIF1A) and endothelial PAS domain protein 1 (EPAS1, best known as HIF2A).^188^ Similarly, ionizing radiation can induce the dedifferentiation of breast cancer cells into CSCs by stimulating the re-expression of pluripotency-associated TFs, which is partly dependent on NOTCH signaling.^189^ In pancreatic cancer, isolation stress leads to the upregulation of lysophosphatidic acid receptor 4 (LPAR4), which promotes transcriptional reprogramming, self-renewal, and tumor initiation by establishing an autonomous tumor-initiating niche.^190^ Examples of reversible differentiation leading to CSC generation and reacquisition of tumorigenic potential have also been reported in AML^191–193^ and melanoma,^194–196^ a setting in which dedifferentiation leads to reacquisition of tumorigenic potential or therapy resistance. Finally, reprogramming to an immature state enables AML cells to evade differentiation therapy,^193^ and melanoma cells exposed to treatment downregulate SOX10, hence limiting SOX10-mediated differentiation in support of chemoresistance.^197^

Some studies have identified epigenetic regulators that actively restore cancer stemness through dedifferentiation. In AML, stem-like reacquisition is associated with altered chromatin accessibility and DNA methylation at differentiation-related genes, often as mediated by DNMT3A and TET2.^198^ Both histone methyltransferases (e.g., PRC2) and demethylases (e.g., KDM1, KDM5, and KDM6 family members) are critical regulators of cancer cell plasticity. For example, oncogenic erb-b2 receptor tyrosine kinase 2 (ERBB2, best known as HER2) signaling upregulates the expression of PRC2 components, including EZH2 and SUZ12 polycomb repressive complex 2 subunit (SUZ12), thus driving epigenetic reprogramming and mammary tumorigenesis.^199^ In breast and prostate cancer, the bone microenvironment promotes reprogramming by activating EZH2, increasing stemness and metastatic potential,^200^ whereas in melanoma, the dynamic expression of KDM5B is correlated with the acquisition of a CSC-like state.^196^ Notably, EZH2 and KDMs also modulate bivalent chromatin domains, i.e., chromatin domains marked by the coexistence of activating (H3K4me1/3) and repressive (H3K27me3) histone modifications.^201^ Bivalency maintains developmental and plasticity-associated genes in a ‘poised’ transcriptional state, allowing for rapid activation or repression in response to environmental or oncogenic cues. Dysregulated activity of these enzymes can disrupt this balance, facilitating aberrant lineage switching and dedifferentiation, thereby contributing to cancer stemness, tumor heterogeneity, and therapy resistance.

Histone methylation modifiers also mediate (immuno)therapy-associated dedifferentiation. In mouse prostate cancer, loss of RB transcriptional corepressor 1 (*Rb1*) and transformation-related protein 53 (*Trp53*) facilitates lineage plasticity and resistance to antiandrogen therapy through upregulation of EZH2 and SOX2, with EZH2 inhibition restoring androgen receptor (AR) expression and treatment sensitivity.^202^ In breast cancer, chemotherapy promotes CSC specification by promoting the recruitment of KDM6A—either alone or in combination with EP300—to pluripotency TF loci, which is promoted by S100 calcium binding protein A10 (S100A10) or adenosine A2b receptor (ADORA2B) signaling and facilitates their transactivation.^109,110^ Recent studies also indicate that immunotherapy can induce epigenetic plasticity in support of a CSC-like state. In melanoma patients who respond to programmed cell death 1 (PDCD, best known as PD-1) blockers, interferon gamma (IFNG, best known as IFN-γ) signaling remodels chromatin to drive neural-crest-like dedifferentiation (i.e., reversion to a more primitive, multipotent state), leading to immunotherapy resistance to both targeted agents and immune checkpoint inhibitors.^203^ Notably, IFN-γ produced by activated T cells has been shown to directly convert non-CSCs into CSCs by inducing BCAT1.^204^ However, whether this effect is linked to the direct inhibition of TET enzymes by BCAT1 activity^45^ remains to be established. Moreover, type I interferon (IFN) released by cancer cells exposed to suboptimal immunogenic chemotherapy activate the histone demethylase KDM1B, triggering a transcriptional rewiring toward a CSC phenotype.^205^ In addition, type I IFN signaling has been shown to repolarize CAFs into a subset that promotes CSC traits through Wnt family member 5 A (WNT5A) paracrine signaling.^206^

Aberrant histone methylation has also been associated with the emergence of drug-tolerant persister (DTP) cells (Fig. 5), a subpopulation of cancer cells that survive therapy by entering a transient, stem-like state due to epigenetic reprogramming, thereby serving as a reservoir for relapse.^207^ In lung cancer and melanoma, KDM5A supports the drug-tolerant phenotype by maintaining a repressed chromatin state.^208,209^ Similarly, KDM5B overexpression in breast cancer increases transcriptomic heterogeneity and contributes to resistance to endocrine therapy, with genetic or pharmacological inhibition of KDM5A/B reducing this heterogeneity, restoring estrogen receptor 1 (ESR1) signaling, and improving therapeutic responses.^210^ In breast cancer, key genes controlling the persister program are held in a “poised” state by bivalent chromatin domains, with KDM6A/B erasing the repressive mark during chemotherapy to promote drug tolerance.^211^ Consequently, KDM6 inhibitors limit DTP cell formation, whereas EZH2 inhibitors favor it. Finally, a subset of slow-cycling GSCs persist upon tyrosine kinase inhibitor (TKI) treatment via chromatin remodeling as driven by KDM6A/B overexpression.^111^ Adding extra layers of complexity, single-cell and barcoding studies have revealed high plasticity and marked heterogeneity among DTP cells across different tumor types during prolonged treatment^212^ and in CML upon extended TKI exposure.^213^ These approaches also demonstrated that therapy directly promotes transcriptional reprogramming and chromatin-mediated plasticity, resulting in DTP cell-dependent residual disease in lung^214^ and breast cancer^215^ patients. These findings point to the complex regulation of drug resistance and persistence involving multiple epigenetic modifiers.

Collectively, these studies demonstrate that epigenetic reprogramming is the major driver of cancer cell dedifferentiation and lineage plasticity, enabling the reacquisition of stem-like traits, resulting in therapeutic resistance, tumor repopulation, and disease progression.

### Epithelial–mesenchymal plasticity

A major example of cell plasticity associated with cancer stemness is the EMT, a process through which epithelial cells lose polarity and adhesion while acquiring mesenchymal features such as motility and invasiveness. The EMT is orchestrated by TFs such as SNAIL, twist family bHLH transcription factor 1 (TWIST1), and zinc finger E-box binding homeobox 1 (ZEB1), which repress epithelial markers such as CDH1 and induce mesenchymal genes such as vimentin (VIM), fibronectin 1 (FN1) and cadherin 2 (CDH2).^216–218^ In addition to promoting invasion and dissemination, the EMT endows cancer cells with CSC-like traits, fostering tumor initiation, therapy resistance, and metastatic potential.^216,217,219–223^

Multiple epigenetic regulators modulate the EMT and contribute to the acquisition of CSC traits, often in a context-dependent manner. For example, DNMT1 and DNMT3A promote the EMT by repressing epithelial genes such as *SNAIL*, at least in HCC via an ARID2-dependent mechanism,^224^ and by silencing epithelial regulators such as CDH1 (in prostate cancer).^225^ Moreover, EZH2 represses EMT-inhibiting genes upon stabilization by SMYD2^226^ or through interaction with SNAIL, as mediated by the long noncoding RNA (lncRNA) HOX transcript antisense RNA (*HOTAIR*).^227^ Likewise, EHMT2 drives the EMT by silencing CDH1 in complex with HDACs,^228^ supporting stemness and invasion in CRC and HCC.^88,228^ WDR5 also sustains the expression of mesenchymal genes (e.g., *VIM*) downstream of TGF-β signaling, and its inhibition suppresses the EMT and enhances sensitivity to chemotherapy.^229^ However, EZH2 can also function as an EMT barrier in specific oncogenic contexts, such as in KRAS-driven lung cancer.^230^

KDM2B supports the EMT as elicited by TGF-β signaling upon repressing epithelial genes such as CDH1, which act in concert with PRC1/PRC2,^231^ whereas KDM1A interacts with SNAI1 to drive EMT-like programs in AML, promoting self-renewal and leukemogenesis.^75^ Notably, in breast cancer, cellular plasticity is facilitated by a bivalent chromatin state at the *ZEB1* promoter in non-CSCs, which allows rapid EMT induction and transition into the CSC state in response to microenvironmental cues such as TGF-β.^232^

Histone acetylation dynamics also play a critical, although context-dependent, role in EMT regulation. For example, HDAC1 represses CDH1 in cooperation with EHMT2 and modulates the splicing of EMT-related transcripts (e.g., SNAI1/2, ZEB1/2) by altering chromatin structure and RNA polymerase II dynamics.^228,233^ Conversely, EP300 cooperates with MYC and DOT1L to transactivate *SNAI1*, *ZEB1*, and *ZEB2* to support the EMT and the acquisition of CSC traits.^127^ In line with this notion, the transient loss of PRC1 components in *Drosophila* epithelial tumor models irreversibly derepresses EMT drivers such as Zn finger homeodomain 1 (zfh1: the ZEB1 ortholog), *de facto* favoring tumorigenesis.^234^

Recent evidence indicates that the EMT is part of a more dynamic process called “epithelial‒mesenchymal plasticity” (EMP), which encompasses a spectrum of intermediate states between the EMT and the reverse process, which is known as “mesenchymal–epithelial transition” (MET).^235^ These hybrid EMT states exhibit increased stemness, plasticity, adaptability to varying microenvironmental conditions and therapeutic pressures, as well as enhanced metastatic potential (Fig. 5).^236–241^

Epigenetic processes play a central role in the EMP and in shaping the hybrid EMT state across cancer types.^242^ Dynamic DNA hypomethylation supports hybrid EMT states in lung cancer, enhancing plasticity, WNT responsiveness, and metastatic dormancy.^243^ The impact of histone methyltransferases on the EMP appears to be tumor dependent. Thus, KMT2C has been reported to limit hybrid EMT states and metastasis in breast cancer, with its loss enhancing IFN-γ signaling and cellular responsiveness to EMP stimuli.^72^ PRC2 acts as a key EMP regulator by repressing mesenchymal genes and stabilizing epithelial identity, at least in some contexts, by cooperating with KMT2D.^244–246^ Notably, PRC2 depletion promotes a quasimesenchymal state linked to enhanced metastasis and poor prognosis.^246^ However, in lung cancer, EZH2 inactivation in the context of FAT atypical cadherin 1 (*FAT1*) loss contributes to a hybrid EMT state, *de facto* increasing stemness and promoting metastatic tumor dissemination.^241^

Together, these findings indicate a central role of epigenetic regulators in orchestrating the EMP, sustaining cancer stemness, metastasis, and therapy resistance.

### CSC plasticity and heterogeneity

Recent evidence suggests that the CSC compartment is composed of distinct subpopulations, each of which is primed for specific functional activities and fates during tumor progression and in response to therapeutic pressures.^17^ Moreover, CSCs are characterized by a high degree of plasticity, dynamically interconverting between CSC states in response to intrinsic cues or external stimuli, including microenvironmental changes and therapy-induced stress (Fig. 5). This plasticity underlies tumor adaptability and resilience, posing a major challenge to durable therapeutic responses.

In HCC, single-cell analyses revealed transcriptionally and functionally distinct CSC subpopulations, each of which has distinct prognostic value.^247^ Moreover, lineage tracing demonstrated that putative liver CSCs exhibit functional diversity in terms of lineage plasticity and dedifferentiation trajectories, supporting tumor growth and heterogeneity.^248^ Similarly, in breast cancer, two distinct CSC types have been identified, which display high interconversion potential in support of local invasion, distant metastatic dissemination, and treatment resistance.^249^

Single-cell transcriptomic and chromatin profiling have also revealed increased heterogeneity in GSCs involving oncogenic, immune, hypoxic, and stemness-related transcriptional programs.^250,251^ Moreover, immunological pressures drive plastic changes in GSCs via epigenetic immunoediting, reprogramming them toward a myeloid-affiliated, immunosuppressive niche.^252^ Likewise, radiotherapy reportedly promotes the transdifferentiation of GSCs into vascular-like cells through EP300-mediated chromatin remodeling, a process that provides trophic support for tumor progression and can be reversed by EP300 inhibition.^253^ In CML, single-cell profiling revealed heterogeneous CSC populations, including therapy-resistant subclones that were detectable at early disease stages.^213^

In CRC, CSC heterogeneity occurs over both time and space. Integrated single-cell transcriptomic and chromatin accessibility profiling across normal, precancerous, and malignant tissues revealed a progressive accumulation of stem-like epithelial cells during tumor initiation, accompanied by aberrant epigenetic and transcriptional reprogramming.^254^ Moreover, the CSC compartment was shown to be controlled (at least in part) by microenvironmental features, with high clonogenic potential being restricted to tumor edges enriched in CAFs.^255^ Finally, under therapeutic pressure, a subset of quiescent CSCs persist and adopt a fetal-like progenitor state to regenerate disease, highlighting the adaptive plasticity of CSCs in response to chemotherapy (at least in the CRC setting).^256^

To conclude, the high degree of plasticity within the CSC compartment enables dynamic state transitions and functional adaptation in response to microenvironmental cues and therapeutic stress.

## Targeting epigenetic modifiers to overcome CSC-related treatment resistance

Targeting epigenetic modifiers has emerged as a promising strategy to eradicate CSCs and overcome resistance to treatment in patients with cancer. This section explores the therapeutic targets, potential, and challenges of modulating epigenetic enzymes in CSCs (Fig. 5).

### DNA methylation

Increased DNMT1, resulting in imbalanced DNA methylation, has been consistently implicated in the tumorigenicity and survival of LSCs, as well as their resistance to treatment.^29,257^ Accordingly, *DNMT1* haploinsufficiency delays leukemogenesis and impairs LSC self-renewal without affecting normal hematopoiesis in murine models of AML, suggesting a therapeutic window for partial DNMT1 suppression.^29^ In line with this, the DNMT1 inhibitor decitabine at low doses has been shown to selectively target LSCs while sparing normal HSCs.^258^ Moreover, DNMT1 inhibition has been reported to increase the sensitivity of CML cells to TKIs.^257^

In addition to hematological malignancies, pharmacological or genetic inhibition of DNMT1 has been shown to eradicate therapy-resistant CSCs in models of breast cancer,^28,259^ CRC,^31^ and GBM.^32^ In the former setting, the CSC-targeting effects of DNMT1 inhibitors appeared to emerge from the concomitant suppression of both DNMT1 and HDAC activity, highlighting the interaction between multiple epigenetic modifiers in the chemoresistant phenotype of CSCs.^259^ Moreover, DNMT1 inhibition has been shown to sensitize GSCs (especially slow-cycling GSCs) to temozolomide, which is often associated with chemoresistance and tumor relapse.^32^ However, whether DNMT1 inhibitors can be safely and effectively combined with temozolomide in patients with GBM remains to be formally investigated.

Although partial or transient DNMT1 inhibition may shrink the CSC compartment without significant hematopoietic toxicity, complete DNMT1 loss leads to progressive HSC exhaustion and multilineage failure, highlighting its essential role in normal stem cell maintenance.^29^ Similarly, DNMT1 deletion in ISCs induces global hypomethylation, loss of crypt architecture, and rapid intestinal failure.^35^ Moreover, APC loss has been shown to perturb DNA methylation and impair stem cell fate in ISCs,^35^ confirming that balanced DNMT1 activity is crucial in sustaining normal tissue homeostasis. Hypomethylating agents such as decitabine and azacitidine display low target specificity, and their clinical use is limited by off-target demethylation, myelosuppression, gastrointestinal toxicity, and frequent relapse (likely due to incomplete CSC eradication).^260^ Thus, novel strategies with improved target selectivity and limited toxicity, ideally within combination regimens, are needed to fully exploit the therapeutic potential of DNMT1 inhibition.

Importantly, therapy-resistant CSCs exhibit unique epigenetic traits that could be exploited therapeutically. For example, LSCs exhibit superior resistance to IDH inhibitors compared with more differentiated AML cells,^261^ possibly due to the elevated prevalence of *IDH1* and *IDH2* mutations in these tumors,^46^ and this has been indicated as a major cause of innate resistance to IDH inhibition in the clinic.^261^ Indeed, *IDH1* mutations appear to establish irreversible epigenetic changes that contribute to stemness and disease progression in both AML^261^ and GBM,^262^ suggesting that epigenetic modifiers specifically targeting these alterations may limit primary resistance to therapy. Similarly, *DNMT3A* mutations have been associated with anthracycline resistance in AML, at least in part, downstream of impaired nucleosome eviction and chromatin remodeling, leading to defective DNA torsional stress repair.^263^ Moreover, leukemias bearing *DNMT3A* mutations exhibit considerable splicing defects, indicating the potential sensitivity of these hematological malignancies to spliceosome-targeting therapies.^264^ The clinical relevance of these findings, however, remains to be validated.

### Histone methylation

H3K-modifying enzymes are emerging as targets to specifically eradicate CSCs or sensitize them to conventional therapeutic approaches. For example, KMT2A has been proposed as a potential target to reduce stemness and overcome therapy resistance in CRC.^60^ Moreover, an in vivo loss-of-function screen based on GBM patient-derived tumor models identified WDR5 and DPY30 as essential for GSC survival, making them promising therapeutic targets for overcoming therapeutic resistance in this oncological setting.^65,67^ However, the role of KMT2A in the preservation of normal stem cells remains debated, particularly in colorectal tissues,^60,63^ necessitating extra caution in the clinical development of KMT2A inhibitors.

Inhibition of KDM1A impairs DNA double-strand break repair, reduces self-renewal, and sensitizes GSCs to temozolomide.^78^ Similarly, destabilizing KDM1A via the GSK3B inhibitor tideglusib sensitizes GBM xenograft models to chemotherapy, *de facto* extending mouse survival.^77^ KDM1A inhibition also exacerbates CSC sensitivity to the TKI sorafenib in HCC.^265,266^ In AML, KDM1A blockers appear to specifically target LSCs without compromising the activity of normal HSCs.^73^ Finally, KDM1B has been reported to promote breast CSC enrichment following suboptimal immunogenic chemotherapy, and KDM1B inhibition sensitizes CSCs to inducers of immunogenic cell death (ICD).^205,267,268^ These findings point to KDM1s as promising targets for eradicating therapy-resistant CSCs across multiple cancer types.

The inhibition of EHMT2 has been shown to sensitize colorectal CSCs to chemo- and radiotherapy by disrupting the DNA damage response.^269^ Moreover, EHMT2 appears to support LSCs in both AML^86^ and CML.^87^ Thus, EHMT2 stands out as a promising therapeutic target to overcome chemoresistance in CSC-associated cancers. That said, EHMT2 expression has also been linked to reduced stemness in preclinical models of lung and skin cancer,^270,271^ suggesting that not all tumors may be equally amenable to EHMT2 inhibition. Notably, EHMT1 has also been shown to contribute to stemness in alveolar rhabdomyosarcoma (a tumor type arising from muscle stem cell transformation), at least in part by promoting the expression of aldehyde dehydrogenase 1 family member A1 (ALDH1A1), which is a key mediator of chemoresistance in CSCs,^272^ suggesting that EHMT1 is a potential target for the development of novel chemosensitizers, at least in some oncological settings.

KDM3B depletion in colorectal CSCs limits tumorigenic potential and chemoresistance by repressing WNT/β-catenin signaling.^93^ Similarly, KDM4A overexpression in breast CSCs appears to create a CSC-specific vulnerability that may be targeted for therapeutic purposes.^273^ Moreover, as both KDM3C and KDM4C play unique roles in LSC maintenance, their inhibition (or inhibition of their molecular target) selectively affects LSC survival without impacting normal hematopoiesis.^97,98^ Intriguingly, KDM3B may also represent a potential therapeutic target for clonal hematopoiesis (a precancerous condition associated with an increased risk for leukemogenesis) by selectively sensitizing *IDH2*/*TET2*-mutant HSCs to Janus kinase 2 (JAK2) inhibitors.^97^

EZH2 is highly expressed by LSCs,^100,101^ making them particularly sensitive to EZH2 inhibition compared with normal HSCs.^101,274^ Similar findings have also been reported in GBM, CRC, and breast cancer.^103,105,107^ Moreover, EZH2 (as well as EZH1) appears to be particularly expressed in chemoresistant, quiescent LSCs compared with their cycling counterparts,^104^ further enhancing its potential as a therapeutic target. That said, at least in the AML setting, *EZH2* loss has also been associated with increased resistance to TKIs, reflecting the activation of a compensatory stemness-supporting pathway involving members of the HOX family.^275^ Moreover, PRC2 (which contains EZH2) has been shown to limit oncogenesis by restraining self-renewal in HSCs,^276^ and countering transformation driven by H3K27 mutations in neural stem cells.^277^

To add further layers of complexity, EZH2 plays a multifaceted role in cancer cell plasticity (see above). Thus, EZH2 not only promotes stemness in multiple tumors, enhancing tumorigenicity, metastatic potential, and therapy resistance,^199,200,202^ but also sustains persistence, at least in breast cancer.^211^ Moreover, the regulatory role of EZH2 in the EMT is highly context-dependent, as EZH2 can act as an EMT promoter or suppressor depending on the cancer type.^226,227,230^ PRC2 is also a key EMP regulator, and its loss or inactivation has been associated with increased metastatic dissemination.^241,246^

The therapeutic targeting of EZH2 presents considerable challenges, with major concerns including limited specificity and scarce cytotoxicity when used as monotherapy, as well as a non-negligible potential for side effects, particularly linked to normal stem cell compartments.^278^ Other complications are related to resistance mechanisms due to compensatory activation of other proteins, such as EZH1,^279^ the complex activity of EZH2 within the PRC2 complex,^102^ the regulatory modulation by lncRNAs, such as HOTAIR,^227,280^ and crosstalk between EZH2 and other epigenetic regulators, such as PRC1.^281^ Emerging evidence supporting the existence of PRC2-independent, noncanonical EZH2 functions further complicates therapeutic approaches.^282^ An improved understanding of the context-dependent roles of EZH2 and its regulatory network is needed to guide the safe and effective use of EZH2 inhibitors in patients with cancer.

Like EZH2, H3K27 demethylases such as KDM6A can either promote or inhibit treatment resistance in CSCs, depending on the oncological setting. On the one hand, KDM6A (as well as KDM6B) hyperactivation has been linked to: (1) TKI resistance in GBM, at least in part by promoting a switch toward reduced cycling,^111^ and (2) chemoresistance in breast CSCs, which involves the epigenetic activation of pluripotency genes.^109,110^ On the other hand, *KDM6A* mutations are common in multiple tumor types^283^ and have been linked with chemoresistance in patients with relapsed AML.^284^ These observations reinforce the notion that multiple epigenetic modifiers influence stemness in a context-dependent manner. Finally, KDM5B depletion reportedly attenuates the AML-suppressing effects of PRC2 inhibitors,^83^ suggesting a complex interaction between EZH2 and chromatin regulators in chemoresistance.

Treatment-resistant CSCs display a unique dependence on DOT1L in multiple cancer types.^121,124,126^ DOT1L is indeed critical for the survival of LSCs but not normal HSCs^121^ or ISCs,^285^ indicating CSC-specific vulnerability with therapeutic potential. Similarly, KDM2B is essential for the maintenance of LSCs^286^ and GSCs,^120^ and its inhibition sensitizes CSCs to chemotherapy.^120^ Future studies will reveal the potential of targeting DOT1L and KDM2B to limit CSC chemoresistance in clinical settings.

The inhibition of PRMT5, which has been implicated in the chemoresistant phenotype of breast CSCs,^129,130^ specifically targets therapy-resistant LSCs.^131^ Similarly, PRMT6 inhibition has been reported to increase GSC radiosensitivity in preclinical GBM models.^138^ However, whether the chemosensitizing effects of PRMT5 or PRMT6 inhibitors on CSCs solely arise from altered histone methylation remains to be formally elucidated, as these agents also have significant effects on posttranscriptional processes.^287,288^

### Histone acetylation and ubiquitination

CRISPR/Cas9- and RNA interference-based screens have identified KAT2A and KAT7 as essential for the maintenance and function of LSCs.^144,146^ Accordingly, KAT2A inhibition promotes LSC differentiation and eradication while sparing HSCs, supporting its specificity as a therapeutic target.^144^ Similarly, KAT7 upregulation as driven by spermidine metabolism has been shown to sustain the clonogenic potential of LSCs, with spermidine restriction limiting LSC function upon KAT7 downregulation in the absence of toxicity to HSCs.^289^ Finally, KAT6A inhibition reportedly depletes LSCs and blocks leukemogenesis, either as a monotherapy or in combination with other AML-differentiating agents.^148^

The HAT EP300 has been implicated in the survival of irradiated GSCs and disease recurrence by promoting their transdifferentiation into cells with vascular-like features,^253^ potentially constituting a therapeutically viable target against CSC chemoresistance. Other HATs, such as KAT2A and its paralog KAT2B, are essential for ISC maintenance, with their loss leading to mitochondrial dysfunction and unsustainable ISC depletion.^290^ Thus, not all HATs are equally suitable targets for depleting CSCs.

Similarly, HDACs exhibit context-dependent effects on stemness, which complicates their exploitation as therapeutic targets. Thus, HDAC1 inhibition has been shown to reduce stemness and restore chemosensitivity in multiple tumor types, including breast cancer, *de facto* synergizing with T-cell therapies in preclinical breast cancer models.^151^ Likewise, pharmacological HDAC6 inhibitors have been reported to synergize with chemotherapy or radiotherapy in preclinical CRC^156^ and GBM^155^ models, respectively, and are generally linked to stemness suppression and restored differentiation. Finally, HDAC3 inhibitors appear to effectively control GSCs in preclinical GBM models, but only in combination with bromodomain containing 4 (BRD4) blockers,^154^ suggesting that (at least in some cases) effectively reversing chemoresistance in the CSC compartment may require combinatorial therapeutic approaches.

Finally, while BMI1 appears to be essential for the maintenance of chemoresistant CSCs in breast cancer,^162^ AML,^169^ and CRC,^165^ such stemness-supporting effects may also benefit normal stem cells, at least in mammary tissues.^162^ However, BMI1 upregulation acts as a driver of mammary and prostate oncogenesis,^161,162^ potentially offering a (perhaps small) window for therapeutic intervention. Indeed, BMI1 inhibition or depletion has been shown to sensitize multiple tumor types to chemotherapy, with minimal short-term toxicity to healthy tissues.^291,292^ However, whether normal stem cells would later suffer from the CSC-targeting effects of BMI1 inhibitors has not been overtly investigated.

Notably, GBMs possess a heterogeneous CSC pool exhibiting distinct transcriptional programs coordinated by either EZH2 or BMI1, suggesting that a combinatorial approach may be required for effective CSC eradication in this oncological setting.^164^ Moreover, the efficacy of BMI1 inhibitors against GSCs appears to be enhanced by the concomitant administration of senolytic agents, reflecting the robust senescent phenotype elicited by BMI1 blockers.^167^ Further investigations are needed to elucidate the actual CSC-eradicating potential of BMI1 inhibitors.

**Fig. 6:**
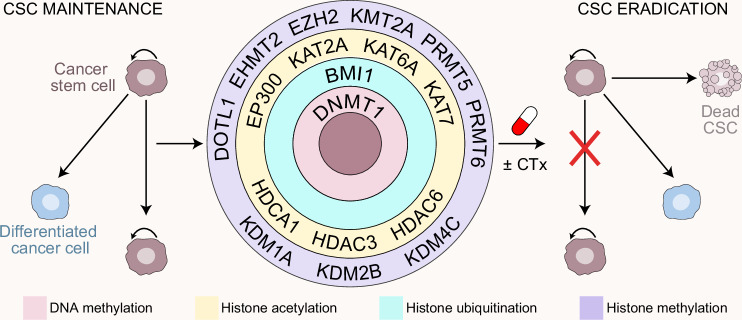
Targeting epigenetic regulators in cancer stem cells. Multiple epigenetic modifiers can be pharmacologically targeted to preferentially eradicate cancer stem cells (CSCs), either as standalone targets or alongside conventional treatments (CTs) as a means to increase therapeutic efficacy. These include enzymes or factors involved in: (1) DNA methylation, such as DNMT1; (2) histone methylation, such as EZH2 or KDM1A; (3) histone acetylation, such as CREBBP, EP300, and multiple histone deacetylases (HDACs); and (4) histone ubiquitination, such as BMI1. However, targeting epigenetic regulators poses key challenges, including toxicity to normal stem cells, the emergence of drug resistance, and incomplete CSC elimination due to tumor plasticity, implying that safe and effective therapeutic strategies may require tumor-specific and combination approaches

In conclusion, targeting epigenetic modifiers that specifically underlie self-renewal and chemoresistance in CSCs represents a promising strategy for improving treatment outcomes across various cancer types (Fig. 6). However, the clinical translation of this approach faces significant challenges, including a nonnegligible potential for toxicity owing to limited specificity (and hence the potential involvement of normal stem cells), as well as limitations of current epigenetic drugs linked to acquired resistance and incomplete CSC eradication. The complex and context-dependent nature of the epigenetic regulation of stemness calls for careful consideration of tumor-specific factors that may influence treatment efficacy in a disease-specific manner and (at least in some cases) for the use of combinatorial therapeutic strategies.

## Concluding remarks

In summary, a variety of epigenetic marks influence the generation and maintenance of CSCs, *de facto* regulating oncogenesis, disease progression, and resistance to therapy for numerous oncological indications. Importantly, some of these DNA and histone modifications (1) control cancer stemness in a consistent manner and (2) are not involved in the preservation of normal stem cells, representing promising targets for the development of novel targeted therapies. In line with this notion, epigenetic drugs, including azacitidine, decitabine, EZH2 blockers and multiple HDAC inhibitors, are currently approved for use in cancer patients, often in malignancies with abundant stem cell compartments, such as hematological tumors.^13,14^ However, a number of questions remain to be answered to unlock the full therapeutic potential of CSC-targeted epigenetic drugs.

First, while epigenetic drugs currently licensed for cancer therapy are active (at least in a fraction of patients), whether such clinical activity specifically emerges from the depletion of the CSC compartment is unclear. Thus, at least in some neoplasms, relatively differentiated malignant cells may also depend on CSC-relevant epigenetic marks, a possibility that requires additional investigation.

Second, at least some epigenetic marks can be deposited or removed by several enzymes of the same family.^293^ In this setting, whether blocking a specific epigenetic modifier is sufficient to mediate antineoplastic effects downstream of CSC dysfunction or whether combinatorial approaches are needed (and if the latter preserves at least some degree of specificity for CSCs) remains to be formally investigated. Moreover, the actual implication of altered epigenetic marks in the CSC-targeting effects of pharmacological or genetic strategies targeting an epigenetic modifier has often been overlooked or addressed in a correlative manner only. Thus, at least some epigenetic modifiers may alter CSC biology through alternative, nonepigenetic mechanisms that have not yet been formally characterized. Notably, several epigenetic modifiers are known to drive CSC immune evasion, indicating that their inhibition can enhance CSC recognition and elimination by the host immune system, potentially sensitizing tumors to (immuno)therapy.^18^

Third, multiple epigenetic marks control transcription in a dynamic and highly interactive manner, at least in part (but not exclusively), reflecting the impact of DNA and histone modifications on chromatin affinity for various epigenetic modifiers.^5^ This implies that the CSC-depleting effects of targeting specific epigenetic traits may actually arise from (or at least involve) a complex crosstalk between distinct epigenetic alterations. In this context, it is worth noting that the accessibility of DNA to TFs and their binding partners is also regulated by global rearrangements of chromatin structure, as catalyzed by ATP-dependent complexes such as the switch/sucrose non-fermentable (SWI/SNF) and the nucleosome remodeling and deacetylase (NuRD) complexes.^294^ Moreover, the conversion of genetic information into cellular functions is further shaped at the posttranscriptional level through mechanisms affecting transcript stability, splicing, export, abundance, and translation, including RNA modifications (e.g., N6-methyladenosine, or m6A), RNA processing events (e.g., alternative splicing, polyadenylation, editing), and regulation by noncoding RNAs such as lncRNAs and microRNAs.^295^ Adding yet another layer of complexity, emerging evidence reveals extensive crosstalk between epitranscriptomics, epigenetics, and chromatin remodeling.^296–298^ For example, histone modifiers regulate m6A marks by modulating the expression of both m6A writers, including methyltransferase 3, N6-adenosine-methyltransferase complex catalytic subunit (METTL3) and METTL14,^299,300^ and m6A erasers, including ALKBH5.^141^ Similarly, lncRNAs regulate EZH2 functions by: (1) guiding its recruitment to specific genomic loci, as in the case of *HOTAIR*^301^ and *HOXA11-AS*;^302^ (2) controlling its posttranslational stability and enzymatic activity, as reported for *ANCR*^303^ and *HOTAIR*;^227,280^ (3) promoting a switch to noncanonical EZH2 activities, as demonstrated for *lncRNA-p21*;^304^ and (4) acting as competing endogenous RNAs to sponge EZH2-targeting microRNAs or its transcriptional regulators, as in the case of *MIAT*^305^ or *HOXD-AS1*.^306^

Finally, the increased heterogeneity and plasticity of the CSC pools imply that CSCs are intrinsically poised to rapidly adapt to therapeutic challenges, including drugs that target epigenetic modifiers. In this context, advanced experimental approaches combining the isolation of CSCs with their identification through cell lineage tracing and clonal barcoding, as well as technologies mapping chromatin accessibility (e.g., scATAC-seq, spatial ATAC-seq, scCAT-seq), profiling histone modifications (e.g., scChIP-seq, scCUT&Tag, spatial CUT&Tag), and analyzing 3D chromatin architecture (e.g., scHi-C) are already redefining our understanding of CSC subpopulations, capturing rare cell states and interactions, and elucidating the mechanisms of CSC-driven heterogeneity, resistance, and tumor evolution. This will guide the development of next-generation epigenetic drugs, potentially requiring the use of combinatorial strategies tailored to address intratumoral CSC diversity.

Despite these and other unresolved questions, it is clear that the epigenetic regulation of transcription plays a critical, although often complex and context-dependent, role in the control of cancer stemness. While considerable work lies ahead, it is tempting to speculate that epigenetic drugs tailored to the CSC compartment may ultimately expand our therapeutic armamentarium against (at least some types of) cancer.

## References

[1] Cramer, P. Organization and regulation of gene transcription. *Nature* **573**, 45–54 (2019). 31462772 10.1038/s41586-019-1517-4

[2] Haberle, V. & Stark, A. Eukaryotic core promoters and the functional basis of transcription initiation. *Nat. Rev. Mol. Cell Biol.* **19**, 621–637 (2018). 29946135 10.1038/s41580-018-0028-8 PMC6205604

[3] Lambert, S. A. et al. The human transcription factors. *Cell* **172**, 650–665 (2018). 29425488 10.1016/j.cell.2018.01.029 PMC12908702

[4] Allis, C. D. & Jenuwein, T. The molecular hallmarks of epigenetic control. *Nat. Rev. Genet.* **17**, 487–500 (2016). 27346641 10.1038/nrg.2016.59

[5] Millán-Zambrano, G., Burton, A., Bannister, A. J. & Schneider, R. Histone post-translational modifications-cause and consequence of genome function. *Nat. Rev. Genet.* **23**, 563–580 (2022). 35338361 10.1038/s41576-022-00468-7

[6] Feinberg, A. P. The key role of epigenetics in human disease prevention and mitigation. *N. Engl. J. Med.* **378**, 1323–1334 (2018). 29617578 10.1056/NEJMra1402513 PMC11567374

[7] Chen, T. & Dent, S. Y. Chromatin modifiers and remodellers: regulators of cellular differentiation. *Nat. Rev. Genet.* **15**, 93–106 (2014). 24366184 10.1038/nrg3607 PMC3999985

[8] Waddington, C. H. The epigenotype. 1942. *Int. J. Epidemiol.* **41**, 10–13 (2012). 22186258 10.1093/ije/dyr184

[9] Elsherbiny, A. & Dobreva, G. Epigenetic memory of cell fate commitment. *Curr. Opin. Cell Biol.* **69**, 80–87 (2021). 33535129 10.1016/j.ceb.2020.12.014

[10] Atlasi, Y. & Stunnenberg, H. G. The interplay of epigenetic marks during stem cell differentiation and development. *Nat. Rev. Genet.* **18**, 643–658 (2017). 28804139 10.1038/nrg.2017.57

[11] Hanahan, D. Hallmarks of cancer: new dimensions. *Cancer Discov.* **12**, 31–46 (2022). 35022204 10.1158/2159-8290.CD-21-1059

[12] Esteller, M. et al. The epigenetic hallmarks of cancer. *Cancer Discov.* **14**, 1783–1809 (2024). 39363741 10.1158/2159-8290.CD-24-0296

[13] Falkenberg, K. J. & Johnstone, R. W. Histone deacetylases and their inhibitors in cancer, neurological diseases and immune disorders. *Nat. Rev. Drug Discov.* **13**, 673–691 (2014). 25131830 10.1038/nrd4360

[14] Quintás-Cardama, A., Santos, F. P. & Garcia-Manero, G. Therapy with azanucleosides for myelodysplastic syndromes. *Nat. Rev. Clin. Oncol.* **7**, 433–444 (2010). 20551943 10.1038/nrclinonc.2010.87

[15] Prager, B. C., Xie, Q., Bao, S. & Rich, J. N. Cancer stem cells: the architects of the tumor ecosystem. *Cell Stem Cell* **24**, 41–53 (2019). 30609398 10.1016/j.stem.2018.12.009 PMC6350931

[16] Batlle, E. & Clevers, H. Cancer stem cells revisited. *Nat. Med.* **23**, 1124–1134 (2017). 28985214 10.1038/nm.4409

[17] Loh, J. J. & Ma, S. Hallmarks of cancer stemness. *Cell Stem Cell* **31**, 617–639 (2024). 38701757 10.1016/j.stem.2024.04.004

[18] Galassi, C., Esteller, M., Vitale, I. & Galluzzi, L. Epigenetic control of immunoevasion in cancer stem cells. *Trends Cancer* **10**, 1052–1071 (2024). 39244477 10.1016/j.trecan.2024.08.004

[19] Galassi, C., Chan, T. A., Vitale, I. & Galluzzi, L. The hallmarks of cancer immune evasion. *Cancer Cell* **42**, 1825–1863 (2024). 39393356 10.1016/j.ccell.2024.09.010

[20] Hall, A. W. et al. Bivalent chromatin domains in glioblastoma reveal a subtype-specific signature of glioma stem cells. *Cancer Res.* **78**, 2463–2474 (2018). 29549165 10.1158/0008-5472.CAN-17-1724 PMC5955797

[21] Easwaran, H. et al. A DNA hypermethylation module for the stem/progenitor cell signature of cancer. *Genome Res.* **22**, 837–849 (2012). 22391556 10.1101/gr.131169.111 PMC3337430

[22] Somervaille, T. C. et al. Hierarchical maintenance of MLL myeloid leukemia stem cells employs a transcriptional program shared with embryonic rather than adult stem cells. *Cell Stem Cell* **4**, 129–140 (2009). 19200802 10.1016/j.stem.2008.11.015 PMC2670853

[23] Ben-Porath, I. et al. An embryonic stem cell-like gene expression signature in poorly differentiated aggressive human tumors. *Nat. Genet* **40**, 499–507 (2008). 18443585 10.1038/ng.127 PMC2912221

[24] Wainwright, E. N. & Scaffidi, P. Epigenetics and cancer stem cells: unleashing, hijacking, and restricting cellular plasticity. *Trends Cancer* **3**, 372–386 (2017). 28718414 10.1016/j.trecan.2017.04.004 PMC5506260

[25] Chu, X. et al. Cancer stem cells: advances in knowledge and implications for cancer therapy. *Signal Transduct. Target Ther.* **9**, 170 (2024). 38965243 10.1038/s41392-024-01851-y PMC11224386

[26] Lee, E. J. et al. Identification of global DNA methylation signatures in glioblastoma-derived cancer stem cells. *J. Genet Genom.* **42**, 355–371 (2015). 10.1016/j.jgg.2015.06.003 PMC4648292 26233891

[27] El Helou, R. et al. Brief reports: a distinct DNA methylation signature defines breast cancer stem cells and predicts cancer outcome. *Stem Cells* **32**, 3031–3036 (2014). 25069843 10.1002/stem.1792

[28] Pathania, R. et al. DNMT1 is essential for mammary and cancer stem cell maintenance and tumorigenesis. *Nat. Commun.* **6**, 6910 (2015). 25908435 10.1038/ncomms7910 PMC4410389

[29] Trowbridge, J. J. et al. Haploinsufficiency of Dnmt1 impairs leukemia stem cell function through derepression of bivalent chromatin domains. *Genes Dev.* **26**, 344–349 (2012). 22345515 10.1101/gad.184341.111 PMC3289882

[30] Liu, H. et al. Downregulation of FOXO3a by DNMT1 promotes breast cancer stem cell properties and tumorigenesis. *Cell Death Differ.* **27**, 966–983 (2020). 31296961 10.1038/s41418-019-0389-3 PMC7206060

[31] Morita, R. et al. DNA methyltransferase 1 is essential for initiation of the colon cancers. *Exp. Mol. Pathol.* **94**, 322–329 (2013). 23064049 10.1016/j.yexmp.2012.10.004

[32] Wei, Y. et al. The interaction between DNMT1 and high-mannose CD133 maintains the slow-cycling state and tumorigenic potential of glioma stem cell. *Adv. Sci.* **9**, e2202216 (2022). 10.1002/advs.202202216 PMC9475542 35798319

[33] Vitale, I. et al. Apoptotic cell death in disease-current understanding of the NCCD 2023. *Cell Death Differ.* **30**, 1097–1154 (2023). 37100955 10.1038/s41418-023-01153-w PMC10130819

[34] Wang, Q. et al. DNMT1-mediated methylation of BEX1 regulates stemness and tumorigenicity in liver cancer. *J. Hepatol.* **75**, 1142–1153 (2021). 34217777 10.1016/j.jhep.2021.06.025

[35] Bruschi, M. et al. Loss of Apc rapidly impairs DNA methylation programs and cell fate decisions in Lgr5(+) intestinal stem cells. *Cancer Res.* **80**, 2101–2113 (2020). 32213541 10.1158/0008-5472.CAN-19-2104

[36] Lopez-Bertoni, H. et al. Sox2 induces glioblastoma cell stemness and tumor propagation by repressing TET2 and deregulating 5hmC and 5mC DNA modifications. *Signal Transduct. Target Ther.* **7**, 37 (2022). 35136034 10.1038/s41392-021-00857-0 PMC8826438

[37] Gkountela, S. et al. Circulating tumor cell clustering shapes DNA methylation to enable metastasis seeding. *Cell* **176**, 98–112.e114 (2019). 30633912 10.1016/j.cell.2018.11.046 PMC6363966

[38] Weissmann, S. et al. Landscape of TET2 mutations in acute myeloid leukemia. *Leukemia* **26**, 934–942 (2012). 22116554 10.1038/leu.2011.326

[39] Tulstrup, M. et al. TET2 mutations are associated with hypermethylation at key regulatory enhancers in normal and malignant hematopoiesis. *Nat. Commun.* **12**, 6061 (2021). 34663818 10.1038/s41467-021-26093-2 PMC8523747

[40] Shih, A. H. et al. Mutational cooperativity linked to combinatorial epigenetic gain of function in acute myeloid leukemia. *Cancer Cell* **27**, 502–515 (2015). 25873173 10.1016/j.ccell.2015.03.009 PMC4518555

[41] Asmar, F. et al. Genome-wide profiling identifies a DNA methylation signature that associates with TET2 mutations in diffuse large B-cell lymphoma. *Haematologica* **98**, 1912–1920 (2013). 23831920 10.3324/haematol.2013.088740 PMC3856967

[42] Guo, L. et al. Perturbing TET2 condensation promotes aberrant genome-wide DNA methylation and curtails leukaemia cell growth. *Nat. Cell Biol.* **26**, 2154–2167 (2024). 39251719 10.1038/s41556-024-01496-7

[43] Cimmino, L. et al. Restoration of TET2 function blocks aberrant self-renewal and leukemia progression. *Cell* **170**, 1079–1095 e1020 (2017). 28823558 10.1016/j.cell.2017.07.032 PMC5755977

[44] Li, Y. et al. TET2-mediated mRNA demethylation regulates leukemia stem cell homing and self-renewal. *Cell Stem Cell* **30**, 1072–1090.e1010 (2023). 37541212 10.1016/j.stem.2023.07.001 PMC11166201

[45] Raffel, S. et al. BCAT1 restricts αKG levels in AML stem cells leading to IDHmut-like DNA hypermethylation. *Nature* **551**, 384–388 (2017). 29144447 10.1038/nature24294

[46] Pirozzi, C. J. & Yan, H. The implications of IDH mutations for cancer development and therapy. *Nat. Rev. Clin. Oncol.* **18**, 645–661 (2021). 34131315 10.1038/s41571-021-00521-0

[47] Figueroa, M. E. et al. Leukemic IDH1 and IDH2 mutations result in a hypermethylation phenotype, disrupt TET2 function, and impair hematopoietic differentiation. *Cancer Cell* **18**, 553–567 (2010). 21130701 10.1016/j.ccr.2010.11.015 PMC4105845

[48] Yao, Q. et al. IDH1 mutation diminishes aggressive phenotype in glioma stem cells. *Int J. Oncol.* **52**, 270–278 (2018). 29115585 10.3892/ijo.2017.4186

[49] Tanabe, R. et al. PRRX1 induced by BMP signaling decreases tumorigenesis by epigenetically regulating glioma-initiating cell properties via DNA methyltransferase 3A. *Mol. Oncol.* **16**, 269–288 (2022). 34214250 10.1002/1878-0261.13051 PMC8732353

[50] Kim, H. et al. Ten-eleven translocation protein 1 modulates medulloblastoma progression. *Genome Biol.* **22**, 125 (2021). 33926529 10.1186/s13059-021-02352-9 PMC8082834

[51] Prasad, P. et al. Hypoxia-mediated epigenetic regulation of stemness in brain tumor cells. *Stem Cells* **35**, 1468–1478 (2017). 28376560 10.1002/stem.2621

[52] Cimmino, L. et al. TET1 is a tumor suppressor of hematopoietic malignancy. *Nat. Immunol.* **16**, 653–662 (2015). 25867473 10.1038/ni.3148 PMC4545281

[53] Huang, H. et al. TET1 plays an essential oncogenic role in MLL-rearranged leukemia. *Proc. Natl. Acad. Sci. USA* **110**, 11994–11999 (2013). 23818607 10.1073/pnas.1310656110 PMC3718141

[54] Chen, Z. et al. Phosphorylation stabilized TET1 acts as an oncoprotein and therapeutic target in B cell acute lymphoblastic leukemia. *Sci. Transl. Med.* **15**, eabq8513 (2023). 36989375 10.1126/scitranslmed.abq8513 PMC11163962

[55] Ley, T. J. et al. DNMT3A mutations in acute myeloid leukemia. *N. Engl. J. Med.* **363**, 2424–2433 (2010). 21067377 10.1056/NEJMoa1005143 PMC3201818

[56] Lu, R. et al. Epigenetic perturbations by Arg882-mutated DNMT3A potentiate aberrant stem cell gene-expression program and acute leukemia development. *Cancer Cell* **30**, 92–107 (2016). 27344947 10.1016/j.ccell.2016.05.008 PMC4945461

[57] Koya, J. et al. DNMT3A R882 mutants interact with polycomb proteins to block haematopoietic stem and leukaemic cell differentiation. *Nat. Commun.* **7**, 10924 (2016). 27010239 10.1038/ncomms10924 PMC4820786

[58] Ferreira, H. J. et al. DNMT3A mutations mediate the epigenetic reactivation of the leukemogenic factor MEIS1 in acute myeloid leukemia. *Oncogene* **35**, 3079–3082 (2016). 26434589 10.1038/onc.2015.359 PMC4705435

[59] Mouti, M. A. et al. KMT2A associates with PHF5A-PHF14-HMG20A-RAI1 subcomplex in pancreatic cancer stem cells and epigenetically regulates their characteristics. *Nat. Commun.* **14**, 5685 (2023). 37709746 10.1038/s41467-023-41297-4 PMC10502114

[60] Grinat, J. et al. The epigenetic regulator Mll1 is required for Wnt-driven intestinal tumorigenesis and cancer stemness. *Nat. Commun.* **11**, 6422 (2020). 33349639 10.1038/s41467-020-20222-z PMC7752919

[61] Godfrey, L. et al. H3K79me2/3 controls enhancer-promoter interactions and activation of the pan-cancer stem cell marker PROM1/CD133 in MLL-AF4 leukemia cells. *Leukemia* **35**, 90–106 (2021). 32242051 10.1038/s41375-020-0808-y PMC7787973

[62] Wang, T. et al. SMYD3 controls a Wnt-responsive epigenetic switch for ASCL2 activation and cancer stem cell maintenance. *Cancer Lett.* **430**, 11–24 (2018). 29746925 10.1016/j.canlet.2018.05.003

[63] Goveas, N. et al. MLL1 is required for maintenance of intestinal stem cells. *PLoS Genet.* **17**, e1009250 (2021). 34860830 10.1371/journal.pgen.1009250 PMC8641872

[64] Macrae, T. A., Fothergill-Robinson, J. & Ramalho-Santos, M. Regulation, functions and transmission of bivalent chromatin during mammalian development. *Nat. Rev. Mol. Cell Biol.* **24**, 6–26 (2023). 36028557 10.1038/s41580-022-00518-2

[65] Mitchell, K. et al. WDR5 represents a therapeutically exploitable target for cancer stem cells in glioblastoma. *Genes Dev.* **37**, 86–102 (2023). 36732025 10.1101/gad.349803.122 PMC10069451

[66] Alvarado, A. G. et al. Glioblastoma cancer stem cells evade innate immune suppression of self-renewal through reduced TLR4 expression. *Cell Stem Cell* **20**, 450–461 e454 (2017). 28089910 10.1016/j.stem.2016.12.001 PMC5822422

[67] Dixit, D. et al. Glioblastoma stem cells reprogram chromatin in vivo to generate selective therapeutic dependencies on DPY30 and phosphodiesterases. *Sci. Transl. Med.* **14**, eabf3917 (2022). 34985972 10.1126/scitranslmed.abf3917

[68] Mak, A. B., Nixon, A. M. & Moffat, J. The mixed lineage leukemia (MLL) fusion-associated gene AF4 promotes CD133 transcription. *Cancer Res* **72**, 1929–1934 (2012). 22337994 10.1158/0008-5472.CAN-11-3589

[69] Nikolic, A. et al. macroH2A2 antagonizes epigenetic programs of stemness in glioblastoma. *Nat. Commun.* **14**, 3062 (2023). 37244935 10.1038/s41467-023-38919-2 PMC10224928

[70] Gallo, M. et al. MLL5 orchestrates a cancer self-renewal state by repressing the histone variant H3.3 and globally reorganizing chromatin. *Cancer Cell* **28**, 715–729 (2015). 26626085 10.1016/j.ccell.2015.10.005

[71] Jiao, Y. et al. The modification role and tumor association with a methyltransferase: KMT2C. *Front. Immunol.* **15**, 1444923 (2024). 39165358 10.3389/fimmu.2024.1444923 PMC11333232

[72] Cui, J. et al. MLL3 loss drives metastasis by promoting a hybrid epithelial-mesenchymal transition state. *Nat. Cell Biol.* **25**, 145–158 (2023). 36604594 10.1038/s41556-022-01045-0 PMC10003829

[73] Harris, W. J. et al. The histone demethylase KDM1A sustains the oncogenic potential of MLL-AF9 leukemia stem cells. *Cancer Cell* **21**, 473–487 (2012). 22464800 10.1016/j.ccr.2012.03.014

[74] Wada, T. et al. Overexpression of the shortest isoform of histone demethylase LSD1 primes hematopoietic stem cells for malignant transformation. *Blood* **125**, 3731–3746 (2015). 25904247 10.1182/blood-2014-11-610907

[75] Carmichael, C. L. et al. The EMT modulator SNAI1 contributes to AML pathogenesis via its interaction with LSD1. *Blood* **136**, 957–973 (2020). 32369597 10.1182/blood.2019002548 PMC7441169

[76] Sareddy, G. R. et al. Novel KDM1A inhibitors induce differentiation and apoptosis of glioma stem cells via unfolded protein response pathway. *Oncogene* **36**, 2423–2434 (2017). 27893719 10.1038/onc.2016.395 PMC5526658

[77] Zhou, A. et al. Nuclear GSK3β promotes tumorigenesis by phosphorylating KDM1A and inducing its deubiquitylation by USP22. *Nat. Cell Biol.* **18**, 954–966 (2016). 27501329 10.1038/ncb3396 PMC5026327

[78] Alejo, S. et al. Lysine-specific histone demethylase 1A (KDM1A/LSD1) inhibition attenuates DNA double-strand break repair and augments the efficacy of temozolomide in glioblastoma. *Neuro. Oncol.* **25**, 1249–1261 (2023). 36652263 10.1093/neuonc/noad018 PMC10326496

[79] Zhang, W. et al. KDM1A promotes thyroid cancer progression and maintains stemness through the Wnt/β-catenin signaling pathway. *Theranostics* **12**, 1500–1517 (2022). 35198054 10.7150/thno.66142 PMC8825597

[80] Lei, Z. J. et al. Lysine-specific demethylase 1 promotes the stemness and chemoresistance of Lgr5(+) liver cancer initiating cells by suppressing negative regulators of β-catenin signaling. *Oncogene* **34**, 3188–3198 (2015). 25893304 10.1038/onc.2015.129

[81] Wu, Y. et al. The deubiquitinase USP28 stabilizes LSD1 and confers stem-cell-like traits to breast cancer cells. *Cell Rep.* **5**, 224–236 (2013). 24075993 10.1016/j.celrep.2013.08.030 PMC4004762

[82] Trempenau, M. L. et al. The histone demethylase KDM5C functions as a tumor suppressor in AML by repression of bivalently marked immature genes. *Leukemia* **37**, 593–605 (2023). 36631623 10.1038/s41375-023-01810-6 PMC9991918

[83] Ren, Z. et al. A PRC2-Kdm5b axis sustains tumorigenicity of acute myeloid leukemia. *Proc. Natl. Acad. Sci. USA* **119**, e2122940119 (2022). 35217626 10.1073/pnas.2122940119 PMC8892512

[84] Chu, Y. et al. SUV39H1 regulates the progression of MLL-AF9-induced acute myeloid leukemia. *Oncogene* **39**, 7239–7252 (2020). 33037410 10.1038/s41388-020-01495-6 PMC7728597

[85] Tan, Y. et al. Matrix softness regulates plasticity of tumour-repopulating cells via H3K9 demethylation and Sox2 expression. *Nat. Commun.* **5**, 4619 (2014). 25099074 10.1038/ncomms5619 PMC4133791

[86] Lehnertz, B. et al. The methyltransferase G9a regulates HoxA9-dependent transcription in AML. *Genes Dev.* **28**, 317–327 (2014). 24532712 10.1101/gad.236794.113 PMC3937511

[87] Zhou, M. et al. Targeting protein lysine methyltransferase G9A impairs self-renewal of chronic myelogenous leukemia stem cells via upregulation of SOX6. *Oncogene* **40**, 3564–3577 (2021). 33931742 10.1038/s41388-021-01799-1

[88] Bergin, C. J. et al. G9a controls pluripotent-like identity and tumor-initiating function in human colorectal cancer. *Oncogene* **40**, 1191–1202 (2021). 33323965 10.1038/s41388-020-01591-7 PMC7878189

[89] Kato, S. et al. Gain-of-function genetic alterations of G9a drive oncogenesis. *Cancer Discov.* **10**, 980–997 (2020). 32269030 10.1158/2159-8290.CD-19-0532 PMC7334057

[90] Fong, K. W. et al. PALI1 promotes tumor growth through competitive recruitment of PRC2 to G9A-target chromatin for dual epigenetic silencing. *Mol. Cell* **82**, 4611–4626.e4617 (2022). 36476474 10.1016/j.molcel.2022.11.010 PMC9812274

[91] Balinth, S. et al. EZH2 regulates a SETDB1/ΔNp63α axis via RUNX3 to drive a cancer stem cell phenotype in squamous cell carcinoma. *Oncogene* **41**, 4130–4144 (2022). 35864175 10.1038/s41388-022-02417-4 PMC10132824

[92] Ramadoss, S. et al. Lysine-specific demethylase KDM3A regulates ovarian cancer stemness and chemoresistance. *Oncogene* **36**, 1537–1545 (2017). 27694900 10.1038/onc.2016.320 PMC5357761

[93] Li, J. et al. KDM3 epigenetically controls tumorigenic potentials of human colorectal cancer stem cells through Wnt/β-catenin signalling. *Nat. Commun.* **8**, 15146 (2017). 28440295 10.1038/ncomms15146 PMC5414094

[94] Chen, Y. et al. Wnt-induced stabilization of KDM4C is required for Wnt/β-Catenin target gene expression and glioblastoma tumorigenesis. *Cancer Res.* **80**, 1049–1063 (2020). 31888886 10.1158/0008-5472.CAN-19-1229 PMC7360480

[95] Liao, T. T. et al. Harnessing stemness and PD-L1 expression by AT-rich interaction domain-containing protein 3B in colorectal cancer. *Theranostics* **10**, 6095–6112 (2020). 32483441 10.7150/thno.44147 PMC7255042

[96] Mallm, J. P. et al. Glioblastoma initiating cells are sensitive to histone demethylase inhibition due to epigenetic deregulation. *Int. J. Cancer* **146**, 1281–1292 (2020). 31456217 10.1002/ijc.32649

[97] Zhu, N. et al. MLL-AF9- and HOXA9-mediated acute myeloid leukemia stem cell self-renewal requires JMJD1C. *J. Clin. Investig.* **126**, 997–1011 (2016). 26878175 10.1172/JCI82978 PMC4767347

[98] Wang, J. et al. Leukemogenic chromatin alterations promote AML leukemia stem cells via a KDM4C-ALKBH5-AXL signaling axis. *Cell Stem Cell* **27**, 81–97.e88 (2020). 32402251 10.1016/j.stem.2020.04.001

[99] Shen, C. et al. RNA demethylase ALKBH5 selectively promotes tumorigenesis and cancer stem cell self-renewal in acute myeloid leukemia. *Cell Stem Cell* **27**, 64–80.e69 (2020). 32402250 10.1016/j.stem.2020.04.009 PMC7335338

[100] Xie, H. et al. Chronic myelogenous leukemia-initiating cells require polycomb group protein EZH2. *Cancer Discov.* **6**, 1237–1247 (2016). 27630126 10.1158/2159-8290.CD-15-1439 PMC5096974

[101] Scott, M. T. et al. Epigenetic reprogramming sensitizes CML stem cells to combined EZH2 and tyrosine kinase inhibition. *Cancer Discov.* **6**, 1248–1257 (2016). 27630125 10.1158/2159-8290.CD-16-0263 PMC6538532

[102] Blackledge, N. P. & Klose, R. J. The molecular principles of gene regulation by polycomb repressive complexes. *Nat. Rev. Mol. Cell Biol.* **22**, 815–833 (2021). 34400841 10.1038/s41580-021-00398-y PMC7612013

[103] Lima-Fernandes, E. et al. Targeting bivalency de-represses Indian Hedgehog and inhibits self-renewal of colorectal cancer-initiating cells. *Nat. Commun.* **10**, 1436 (2019). 30926792 10.1038/s41467-019-09309-4 PMC6441108

[104] Fujita, S. et al. Dual inhibition of EZH1/2 breaks the quiescence of leukemia stem cells in acute myeloid leukemia. *Leukemia* **32**, 855–864 (2018). 28951561 10.1038/leu.2017.300

[105] Kim, E. et al. Phosphorylation of EZH2 activates STAT3 signaling via STAT3 methylation and promotes tumorigenicity of glioblastoma stem-like cells. *Cancer Cell* **23**, 839–852 (2013). 23684459 10.1016/j.ccr.2013.04.008 PMC4109796

[106] Suvà, M. L. et al. EZH2 is essential for glioblastoma cancer stem cell maintenance. *Cancer Res.* **69**, 9211–9218 (2009). 19934320 10.1158/0008-5472.CAN-09-1622

[107] Chang, C. J. et al. EZH2 promotes expansion of breast tumor initiating cells through activation of RAF1-β-catenin signaling. *Cancer Cell* **19**, 86–100 (2011). 21215703 10.1016/j.ccr.2010.10.035 PMC3041516

[108] LaBella, K. A. et al. Telomere dysfunction alters intestinal stem cell dynamics to promote cancer. *Dev. Cell* **59**, 1475–1486.e1475 (2024). 38574731 10.1016/j.devcel.2024.03.020 PMC11379129

[109] Lu, H. et al. Chemotherapy-induced S100A10 recruits KDM6A to facilitate OCT4-mediated breast cancer stemness. *J. Clin. Investig.* **130**, 4607–4623 (2020). 32427586 10.1172/JCI138577 PMC7456215

[110] Lan, J. et al. Chemotherapy-induced adenosine A2B receptor expression mediates epigenetic regulation of pluripotency factors and promotes breast cancer stemness. *Theranostics* **12**, 2598–2612 (2022). 35401817 10.7150/thno.70581 PMC8965495

[111] Liau, B. B. et al. Adaptive chromatin remodeling drives glioblastoma stem cell plasticity and drug tolerance. *Cell Stem Cell* **20**, 233–246.e237 (2017). 27989769 10.1016/j.stem.2016.11.003 PMC5291795

[112] Zhou, Y. et al. Setd2 regulates quiescence and differentiation of adult hematopoietic stem cells by restricting RNA polymerase II elongation. *Haematologica* **103**, 1110–1123 (2018). 29650642 10.3324/haematol.2018.187708 PMC6029524

[113] Zhang, Y. L. et al. Setd2 deficiency impairs hematopoietic stem cell self-renewal and causes malignant transformation. *Cell Res.* **28**, 476–490 (2018). 29531312 10.1038/s41422-018-0015-9 PMC5939047

[114] Zhu, X. et al. Identification of functional cooperative mutations of SETD2 in human acute leukemia. *Nat. Genet.* **46**, 287–293 (2014). 24509477 10.1038/ng.2894 PMC4440318

[115] Zhu, L. et al. ASH1L links histone H3 lysine 36 dimethylation to MLL leukemia. *Cancer Discov.* **6**, 770–783 (2016). 27154821 10.1158/2159-8290.CD-16-0058 PMC4930721

[116] Ren, J. et al. Histone methyltransferase WHSC1 loss dampens MHC-I antigen presentation pathway to impair IFN-gamma-stimulated antitumor immunity. *J. Clin. Investig.* **132**, e153167 (2022). 35230972 10.1172/JCI153167 PMC9012282

[117] Saloura, V. et al. WHSC1 monomethylates histone H1 and induces stem-cell like features in squamous cell carcinoma of the head and neck. *Neoplasia* **22**, 283–293 (2020). 32497898 10.1016/j.neo.2020.05.002 PMC7265065

[118] Jeong, G. Y. et al. NSD3-induced methylation of H3K36 activates NOTCH signaling to drive breast tumor initiation and metastatic progression. *Cancer Res.* **81**, 77–90 (2021). 32967925 10.1158/0008-5472.CAN-20-0360

[119] Lin, Q. et al. ZHX2 restricts hepatocellular carcinoma by suppressing stem cell-like traits through KDM2A-mediated H3K36 demethylation. *EBioMedicine* **53**, 102676 (2020). 32114388 10.1016/j.ebiom.2020.102676 PMC7047184

[120] Staberg, M. et al. Targeting glioma stem-like cell survival and chemoresistance through inhibition of lysine-specific histone demethylase KDM2B. *Mol. Oncol.* **12**, 406–420 (2018). 29360266 10.1002/1878-0261.12174 PMC5830623

[121] Bernt, K. M. et al. MLL-rearranged leukemia is dependent on aberrant H3K79 methylation by DOT1. *L. Cancer Cell.* **20**, 66–78 (2011). 21741597 10.1016/j.ccr.2011.06.010 PMC3329803

[122] He, C. et al. Histone methyltransferase NSD2 regulates apoptosis and chemosensitivity in osteosarcoma. *Cell Death Dis.* **10**, 65 (2019). 30683853 10.1038/s41419-019-1347-1 PMC6347630

[123] Wang, G. et al. The DNA damage-independent ATM signalling maintains CBP/DOT1L axis in MLL rearranged acute myeloid leukaemia. *Oncogene* **43**, 1900–1916 (2024). 38671157 10.1038/s41388-024-02998-2 PMC11178498

[124] MacLeod, G. et al. Genome-wide CRISPR-Cas9 screens expose genetic vulnerabilities and mechanisms of temozolomide sensitivity in glioblastoma stem cells. *Cell Rep.* **27**, 971–986.e979 (2019). 30995489 10.1016/j.celrep.2019.03.047

[125] Li, P., Zhang, Z. & Sun, P. DOT1L promotes expression of CD44 through the Wnt/β-catenin signaling pathway in early gastric carcinoma. *J. Cancer* **15**, 2276–2291 (2024). 38495505 10.7150/jca.90170 PMC10937288

[126] Kurani, H. et al. DOT1L is a novel cancer stem cell target for triple-negative breast cancer. *Clin. Cancer Res.* **28**, 1948–1965 (2022). 35135840 10.1158/1078-0432.CCR-21-1299 PMC9365344

[127] Cho, M. H. et al. DOT1L cooperates with the c-Myc-p300 complex to epigenetically derepress CDH1 transcription factors in breast cancer progression. *Nat. Commun.* **6**, 7821 (2015). 26199140 10.1038/ncomms8821 PMC4525167

[128] Xu, J. & Richard, S. Cellular pathways influenced by protein arginine methylation: implications for cancer. *Mol. Cell* **81**, 4357–4368 (2021). 34619091 10.1016/j.molcel.2021.09.011 PMC8571027

[129] Chiang, K. et al. PRMT5 is a critical regulator of breast cancer stem cell function via histone methylation and FOXP1 expression. *Cell Rep.* **21**, 3498–3513 (2017). 29262329 10.1016/j.celrep.2017.11.096 PMC5746596

[130] Wang, Z. et al. PRMT5 determines the sensitivity to chemotherapeutics by governing stemness in breast cancer. *Breast Cancer Res. Treat.* **168**, 531–542 (2018). 29185119 10.1007/s10549-017-4597-6

[131] Jin, Y. et al. Targeting methyltransferase PRMT5 eliminates leukemia stem cells in chronic myelogenous leukemia. *J. Clin. Investig.* **126**, 3961–3980 (2016). 27643437 10.1172/JCI85239 PMC5096815

[132] Li, Y. et al. PRMT5 is required for lymphomagenesis triggered by multiple oncogenic drivers. *Cancer Discov.* **5**, 288–303 (2015). 25582697 10.1158/2159-8290.CD-14-0625 PMC4355177

[133] Dong, H. et al. Targeting PRMT9-mediated arginine methylation suppresses cancer stem cell maintenance and elicits cGAS-mediated anticancer immunity. *Nat. Cancer* **5**, 601–624 (2024). 38413714 10.1038/s43018-024-00736-x PMC11056319

[134] Cheng, Y. et al. Decoding m(6)A RNA methylome identifies PRMT6-regulated lipid transport promoting AML stem cell maintenance. *Cell Stem Cell* **30**, 69–85.e67 (2023). 36574771 10.1016/j.stem.2022.12.003

[135] Liu, C. et al. Loss of PRMT7 reprograms glycine metabolism to selectively eradicate leukemia stem cells in CML. *Cell Metab.* **34**, 818–835.e817 (2022). 35508169 10.1016/j.cmet.2022.04.004

[136] Dolezal, E. et al. The BTG2-PRMT1 module limits pre-B cell expansion by regulating the CDK4-Cyclin-D3 complex. *Nat. Immunol.* **18**, 911–920 (2017). 28628091 10.1038/ni.3774

[137] Liao, Y. et al. PRMT3 drives glioblastoma progression by enhancing HIF1A and glycolytic metabolism. *Cell Death Dis.* **13**, 943 (2022). 36351894 10.1038/s41419-022-05389-1 PMC9646854

[138] Huang, T. et al. PRMT6 methylation of RCC1 regulates mitosis, tumorigenicity, and radiation response of glioblastoma stem cells. *Mol. Cell* **81**, 1276–1291.e1279 (2021). 33539787 10.1016/j.molcel.2021.01.015 PMC7979509

[139] Dong, F. et al. PRMT2 links histone H3R8 asymmetric dimethylation to oncogenic activation and tumorigenesis of glioblastoma. *Nat. Commun.* **9**, 4552 (2018). 30382083 10.1038/s41467-018-06968-7 PMC6208368

[140] Tao, W. et al. SATB2 drives glioblastoma growth by recruiting CBP to promote FOXM1 expression in glioma stem cells. *EMBO Mol. Med.* **12**, e12291 (2020). 33124191 10.15252/emmm.202012291 PMC7721366

[141] Hao, L. et al. ALKBH5-mediated m(6)A demethylation of FOXM1 mRNA promotes progression of uveal melanoma. *Aging* **13**, 4045–4062 (2021). 33428593 10.18632/aging.202371 PMC7906204

[142] Jian, Y. et al. Jade family PHD finger 3 (JADE3) increases cancer stem cell-like properties and tumorigenicity in colon cancer. *Cancer Lett.* **428**, 1–11 (2018). 29660380 10.1016/j.canlet.2018.04.012

[143] Chang, C. H. et al. The pRb/RBL2-E2F1/4-GCN5 axis regulates cancer stem cell formation and G0 phase entry/exit by paracrine mechanisms. *Nat. Commun.* **15**, 3580 (2024). 38678032 10.1038/s41467-024-47680-z PMC11055877

[144] Tzelepis, K. et al. A CRISPR dropout screen identifies genetic vulnerabilities and therapeutic targets in acute myeloid leukemia. *Cell Rep.* **17**, 1193–1205 (2016). 27760321 10.1016/j.celrep.2016.09.079 PMC5081405

[145] Cabal-Hierro, L. et al. Chromatin accessibility promotes hematopoietic and leukemia stem cell activity. *Nat. Commun.* **11**, 1406 (2020). 32179749 10.1038/s41467-020-15221-z PMC7076002

[146] MacPherson, L. et al. HBO1 is required for the maintenance of leukaemia stem cells. *Nature* **577**, 266–270 (2020). 31827282 10.1038/s41586-019-1835-6

[147] Li, J. et al. ZMYND11-MBTD1 induces leukemogenesis through hijacking NuA4/TIP60 acetyltransferase complex and a PWWP-mediated chromatin association mechanism. *Nat. Commun.* **12**, 1045 (2021). 33594072 10.1038/s41467-021-21357-3 PMC7886901

[148] Yan, F. et al. KAT6A and ENL form an epigenetic transcriptional control module to drive critical leukemogenic gene-expression programs. *Cancer Discov.* **12**, 792–811 (2022). 34853079 10.1158/2159-8290.CD-20-1459 PMC8916037

[149] Domingues, A. F. et al. Loss of Kat2a enhances transcriptional noise and depletes acute myeloid leukemia stem-like cells. *Elife* **9**, e51754 (2020). 31985402 10.7554/eLife.51754 PMC7039681

[150] Pan, F. et al. Enhancer remodeling drives MLL oncogene-dependent transcriptional dysregulation in leukemia stem cells. *Blood Adv.* **7**, 2504–2519 (2023). 36705973 10.1182/bloodadvances.2022008787 PMC10248086

[151] Song, K. H. et al. HDAC1 upregulation by NANOG promotes multidrug resistance and a stem-like phenotype in immune edited tumor cells. *Cancer Res.* **77**, 5039–5053 (2017). 28716899 10.1158/0008-5472.CAN-17-0072 PMC8171587

[152] Lo Cascio, C. et al. Nonredundant, isoform-specific roles of HDAC1 in glioma stem cells. *JCI Insight* **6**, e149232 (2021). 34494550 10.1172/jci.insight.149232 PMC8492336

[153] Bahia, R. K. et al. Epigenetic and molecular coordination between HDAC2 and SMAD3-SKI regulates essential brain tumour stem cell characteristics. *Nat. Commun.* **14**, 5051 (2023). 37598220 10.1038/s41467-023-40776-y PMC10439933

[154] Wang, Q. et al. A combination of BRD4 and HDAC3 inhibitors synergistically suppresses glioma stem cell growth by blocking GLI1/IL6/STAT3 signaling axis. *Mol. Cancer Ther.* **19**, 2542–2553 (2020). 32999044 10.1158/1535-7163.MCT-20-0037

[155] Yang, W. et al. HDAC6 inhibition induces glioma stem cells differentiation and enhances cellular radiation sensitivity through the SHH/Gli1 signaling pathway. *Cancer Lett.* **415**, 164–176 (2018). 29222038 10.1016/j.canlet.2017.12.005

[156] Wang, T. et al. The inflammatory cytokine IL-6 induces FRA1 deacetylation promoting colorectal cancer stem-like properties. *Oncogene* **38**, 4932–4947 (2019). 30804456 10.1038/s41388-019-0763-0 PMC6756002

[157] Liu, L. et al. SIRT1-mediated transcriptional regulation of SOX2 is important for self-renewal of liver cancer stem cells. *Hepatology* **64**, 814–827 (2016). 27312708 10.1002/hep.28690

[158] Bi, L. et al. HDAC11 regulates glycolysis through the LKB1/AMPK signaling pathway to maintain hepatocellular carcinoma stemness. *Cancer Res.* **81**, 2015–2028 (2021). 33602787 10.1158/0008-5472.CAN-20-3044

[159] Sun, J. et al. SIRT1 activation disrupts maintenance of myelodysplastic syndrome stem and progenitor cells by restoring TET2 function. *Cell Stem Cell* **23**, 355–369.e359 (2018). 30146412 10.1016/j.stem.2018.07.018 PMC6143172

[160] Singh, S. K. et al. Sirt1 ablation promotes stress-induced loss of epigenetic and genomic hematopoietic stem and progenitor cell maintenance. *J. Exp. Med.* **210**, 987–1001 (2013). 23630229 10.1084/jem.20121608 PMC3646499

[161] Lukacs, R. U., Memarzadeh, S., Wu, H. & Witte, O. N. Bmi-1 is a crucial regulator of prostate stem cell self-renewal and malignant transformation. *Cell Stem Cell* **7**, 682–693 (2010). 21112563 10.1016/j.stem.2010.11.013 PMC3019762

[162] Liu, S. et al. Hedgehog signaling and Bmi-1 regulate self-renewal of normal and malignant human mammary stem cells. *Cancer Res.* **66**, 6063–6071 (2006). 16778178 10.1158/0008-5472.CAN-06-0054 PMC4386278

[163] Lessard, J. & Sauvageau, G. Bmi-1 determines the proliferative capacity of normal and leukaemic stem cells. *Nature* **423**, 255–260 (2003). 12714970 10.1038/nature01572

[164] Jin, X. et al. Targeting glioma stem cells through combined BMI1 and EZH2 inhibition. *Nat. Med.* **23**, 1352–1361 (2017). 29035367 10.1038/nm.4415 PMC5679732

[165] Kreso, A. et al. Self-renewal as a therapeutic target in human colorectal cancer. *Nat. Med.* **20**, 29–36 (2014). 24292392 10.1038/nm.3418

[166] Chung, C. Y. et al. Cbx8 acts non-canonically with Wdr5 to promote mammary tumorigenesis. *Cell Rep.* **16**, 472–486 (2016). 27346354 10.1016/j.celrep.2016.06.002 PMC4972459

[167] Balakrishnan, I. et al. Senescence induced by BMI1 inhibition is a therapeutic vulnerability in H3K27M-mutant DIPG. *Cell Rep.* **33**, 108286 (2020). 33086074 10.1016/j.celrep.2020.108286 PMC7574900

[168] Klauke, K. et al. Polycomb Cbx family members mediate the balance between haematopoietic stem cell self-renewal and differentiation. *Nat. Cell Biol.* **15**, 353–362 (2013). 23502315 10.1038/ncb2701

[169] Yuan, J. et al. Bmi1 is essential for leukemic reprogramming of myeloid progenitor cells. *Leukemia* **25**, 1335–1343 (2011). 21527932 10.1038/leu.2011.85

[170] Tan, J. et al. CBX8, a polycomb group protein, is essential for MLL-AF9-induced leukemogenesis. *Cancer Cell* **20**, 563–575 (2011). 22094252 10.1016/j.ccr.2011.09.008 PMC3220883

[171] Jung, J. et al. CBX7 induces self-renewal of human normal and malignant hematopoietic stem and progenitor cells by canonical and non-canonical interactions. *Cell Rep.* **26**, 1906–1918.e1908 (2019). 30759399 10.1016/j.celrep.2019.01.050

[172] Shima, H. et al. Ring1A and Ring1B inhibit expression of Glis2 to maintain murine MOZ-TIF2 AML stem cells. *Blood* **131**, 1833–1845 (2018). 29371181 10.1182/blood-2017-05-787226

[173] Mehta, A. & Stanger, B. Z. Lineage plasticity: the new cancer hallmark on the block. *Cancer Res.* **84**, 184–191 (2024). 37963209 10.1158/0008-5472.CAN-23-1067 PMC10841583

[174] Qin, X. & Tape, C. J. Functional analysis of cell plasticity using single-cell technologies. *Trends Cell Biol.* **34**, 854–864 (2024). 38355348 10.1016/j.tcb.2024.01.006

[175] Yuan, S., Norgard, R. J. & Stanger, B. Z. Cellular plasticity in cancer. *Cancer Discov.* **9**, 837–851 (2019). 30992279 10.1158/2159-8290.CD-19-0015 PMC6606363

[176] Vitale, I., Shema, E., Loi, S. & Galluzzi, L. Intratumoral heterogeneity in cancer progression and response to immunotherapy. *Nat. Med.* **27**, 212–224 (2021). 33574607 10.1038/s41591-021-01233-9

[177] Dhimolea, E. et al. An embryonic diapause-like adaptation with suppressed Myc activity enables tumor treatment persistence. *Cancer Cell* **39**, 240–256.e211 (2021). 33417832 10.1016/j.ccell.2020.12.002 PMC8670073

[178] Laughney, A. M. et al. Regenerative lineages and immune-mediated pruning in lung cancer metastasis. *Nat. Med.* **26**, 259–269 (2020). 32042191 10.1038/s41591-019-0750-6 PMC7021003

[179] Rehman, S. K. et al. Colorectal cancer cells enter a diapause-like DTP state to survive chemotherapy. *Cell* **184**, 226–242.e221 (2021). 33417860 10.1016/j.cell.2020.11.018 PMC8437243

[180] de Sousa e Melo, F. et al. A distinct role for Lgr5(+) stem cells in primary and metastatic colon cancer. *Nature* **543**, 676–680 (2017). 28358093 10.1038/nature21713

[181] Fumagalli, A. et al. Plasticity of Lgr5-negative cancer cells drives metastasis in colorectal cancer. *Cell Stem Cell* **26**, 569–578.e567 (2020). 32169167 10.1016/j.stem.2020.02.008 PMC7118369

[182] Shimokawa, M. et al. Visualization and targeting of LGR5(+) human colon cancer stem cells. *Nature* **545**, 187–192 (2017). 28355176 10.1038/nature22081

[183] Juul, N. H. et al. KRAS(G12D) drives lepidic adenocarcinoma through stem-cell reprogramming. *Nature* **619**, 860–867 (2023). 37468622 10.1038/s41586-023-06324-w PMC10423036

[184] Schwitalla, S. et al. Intestinal tumorigenesis initiated by dedifferentiation and acquisition of stem-cell-like properties. *Cell* **152**, 25–38 (2013). 23273993 10.1016/j.cell.2012.12.012

[185] Todaro, M. et al. CD44v6 is a marker of constitutive and reprogrammed cancer stem cells driving colon cancer metastasis. *Cell Stem Cell* **14**, 342–356 (2014). 24607406 10.1016/j.stem.2014.01.009

[186] Vermeulen, L. et al. Wnt activity defines colon cancer stem cells and is regulated by the microenvironment. *Nat. Cell Biol.* **12**, 468–476 (2010). 20418870 10.1038/ncb2048

[187] Dirkse, A. et al. Stem cell-associated heterogeneity in Glioblastoma results from intrinsic tumor plasticity shaped by the microenvironment. *Nat. Commun.* **10**, 1787 (2019). 30992437 10.1038/s41467-019-09853-z PMC6467886

[188] Lee, G. et al. Dedifferentiation of glioma cells to glioma stem-like cells by therapeutic stress-induced HIF signaling in the recurrent GBM model. *Mol. Cancer Ther.* **15**, 3064–3076 (2016). 27765847 10.1158/1535-7163.MCT-15-0675 PMC5298928

[189] Lagadec, C. et al. Radiation-induced reprogramming of breast cancer cells. *Stem Cells* **30**, 833–844 (2012). 22489015 10.1002/stem.1058 PMC3413333

[190] Wu, C. et al. Pancreatic cancer cells upregulate LPAR4 in response to isolation stress to promote an ECM-enriched niche and support tumour initiation. *Nat. Cell Biol.* **25**, 309–322 (2023). 36646789 10.1038/s41556-022-01055-y PMC10280815

[191] Agarwal, A. et al. Differentiation of leukemic blasts is not completely blocked in acute myeloid leukemia. *Proc. Natl. Acad. Sci. USA* **116**, 24593–24599 (2019). 31754026 10.1073/pnas.1904091116 PMC6900505

[192] Chao, M. P. et al. Human AML-iPSCs reacquire leukemic properties after differentiation and model clonal variation of disease. *Cell Stem Cell* **20**, 329–344.e327 (2017). 28089908 10.1016/j.stem.2016.11.018 PMC5508733

[193] McKenzie, M. D. et al. Interconversion between tumorigenic and differentiated states in acute myeloid leukemia. *Cell Stem Cell* **25**, 258–272.e259 (2019). 31374198 10.1016/j.stem.2019.07.001

[194] Köhler, C. et al. Mouse cutaneous melanoma induced by mutant braf arises from expansion and dedifferentiation of mature pigmented melanocytes. *Cell Stem Cell* **21**, 679–693.e676 (2017). 29033351 10.1016/j.stem.2017.08.003

[195] Quintana, E. et al. Phenotypic heterogeneity among tumorigenic melanoma cells from patients that is reversible and not hierarchically organized. *Cancer Cell* **18**, 510–523 (2010). 21075313 10.1016/j.ccr.2010.10.012 PMC3031091

[196] Roesch, A. et al. A temporarily distinct subpopulation of slow-cycling melanoma cells is required for continuous tumor growth. *Cell* **141**, 583–594 (2010). 20478252 10.1016/j.cell.2010.04.020 PMC2882693

[197] Shaffer, S. M. et al. Rare cell variability and drug-induced reprogramming as a mode of cancer drug resistance. *Nature* **546**, 431–435 (2017). 28607484 10.1038/nature22794 PMC5542814

[198] Wang, W. et al. RETRACTED ARTICLE: inhibition of miR-296-5p protects the heart from cardiac hypertrophy by targeting CACNG6. *Gene Ther.* **28**, 391 (2021). 31844153 10.1038/s41434-019-0109-0 PMC8221998

[199] Smith, H. W. et al. An ErbB2/c-Src axis links bioenergetics with PRC2 translation to drive epigenetic reprogramming and mammary tumorigenesis. *Nat. Commun.* **10**, 2901 (2019). 31263101 10.1038/s41467-019-10681-4 PMC6603039

[200] Zhang, W. et al. The bone microenvironment invigorates metastatic seeds for further dissemination. *Cell* **184**, 2471–2486.e2420 (2021). 33878291 10.1016/j.cell.2021.03.011 PMC8087656

[201] Blanco, E. et al. The bivalent genome: characterization, structure, and regulation. *Trends Genet.* **36**, 118–131 (2020). 31818514 10.1016/j.tig.2019.11.004

[202] Ku, S. Y. et al. Rb1 and Trp53 cooperate to suppress prostate cancer lineage plasticity, metastasis, and antiandrogen resistance. *Science* **355**, 78–83 (2017). 28059767 10.1126/science.aah4199 PMC5367887

[203] Kim, Y. J. et al. Melanoma dedifferentiation induced by IFN-γ epigenetic remodeling in response to anti-PD-1 therapy. *J. Clin. Invest* **131**, e145859 (2021). 33914706 10.1172/JCI145859 PMC8203459

[204] Beziaud, L. et al. IFNgamma-induced stem-like state of cancer cells as a driver of metastatic progression following immunotherapy. *Cell Stem Cell* **30**, 818–831 e816 (2023). 37267916 10.1016/j.stem.2023.05.007

[205] Musella, M. et al. Type I IFNs promote cancer cell stemness by triggering the epigenetic regulator KDM1B. *Nat. Immunol.* **23**, 1379–1392 (2022). 36002648 10.1038/s41590-022-01290-3 PMC9477743

[206] Ma, Z. et al. Interferon-dependent SLC14A1(+) cancer-associated fibroblasts promote cancer stemness via WNT5A in bladder cancer. *Cancer Cell* **40**, 1550–1565.e1557 (2022). 36459995 10.1016/j.ccell.2022.11.005

[207] Russo, M. et al. Cancer drug-tolerant persister cells: from biological questions to clinical opportunities. *Nat. Rev. Cancer* **24**, 694–717 (2024). 39223250 10.1038/s41568-024-00737-z PMC12622869

[208] Sharma, S. V. et al. A chromatin-mediated reversible drug-tolerant state in cancer cell subpopulations. *Cell* **141**, 69–80 (2010). 20371346 10.1016/j.cell.2010.02.027 PMC2851638

[209] Vinogradova, M. et al. An inhibitor of KDM5 demethylases reduces survival of drug-tolerant cancer cells. *Nat. Chem. Biol.* **12**, 531–538 (2016). 27214401 10.1038/nchembio.2085

[210] Hinohara, K. et al. KDM5 histone demethylase activity links cellular transcriptomic heterogeneity to therapeutic resistance. *Cancer Cell* **34**, 939–953.e939 (2018). 30472020 10.1016/j.ccell.2018.10.014 PMC6310147

[211] Marsolier, J. et al. H3K27me3 conditions chemotolerance in triple-negative breast cancer. *Nat. Genet* **54**, 459–468 (2022). 35410383 10.1038/s41588-022-01047-6 PMC7612638

[212] Oren, Y. et al. Cycling cancer persister cells arise from lineages with distinct programs. *Nature* **596**, 576–582 (2021). 34381210 10.1038/s41586-021-03796-6 PMC9209846

[213] Giustacchini, A. et al. Single-cell transcriptomics uncovers distinct molecular signatures of stem cells in chronic myeloid leukemia. *Nat. Med.* **23**, 692–702 (2017). 28504724 10.1038/nm.4336

[214] Maynard, A. et al. Therapy-induced evolution of human lung cancer revealed by single-cell RNA sequencing. *Cell* **182**, 1232–1251.e1222 (2020). 32822576 10.1016/j.cell.2020.07.017 PMC7484178

[215] Kim, C. et al. Chemoresistance evolution in triple-negative breast cancer delineated by single-cell sequencing. *Cell* **173**, 879–893.e813 (2018). 29681456 10.1016/j.cell.2018.03.041 PMC6132060

[216] Dongre, A. & Weinberg, R. A. New insights into the mechanisms of epithelial-mesenchymal transition and implications for cancer. *Nat. Rev. Mol. Cell Biol.* **20**, 69–84 (2019). 30459476 10.1038/s41580-018-0080-4

[217] Lambert, A. W. & Weinberg, R. A. Linking EMT programmes to normal and neoplastic epithelial stem cells. *Nat. Rev. Cancer* **21**, 325–338 (2021). 33547455 10.1038/s41568-021-00332-6

[218] Yang, J. et al. Author correction: guidelines and definitions for research on epithelial-mesenchymal transition. *Nat. Rev. Mol. Cell Biol.* **22**, 834 (2021). 34654908 10.1038/s41580-021-00428-9 PMC8604719

[219] Yang, J. et al. Guidelines and definitions for research on epithelial-mesenchymal transition. *Nat. Rev. Mol. Cell Biol.* **21**, 341–352 (2020). 32300252 10.1038/s41580-020-0237-9 PMC7250738

[220] Guo, W. et al. Slug and Sox9 cooperatively determine the mammary stem cell state. *Cell* **148**, 1015–1028 (2012). 22385965 10.1016/j.cell.2012.02.008 PMC3305806

[221] Mani, S. A. et al. The epithelial-mesenchymal transition generates cells with properties of stem cells. *Cell* **133**, 704–715 (2008). 18485877 10.1016/j.cell.2008.03.027 PMC2728032

[222] Terekhanova, N. V. et al. Epigenetic regulation during cancer transitions across 11 tumour types. *Nature* **623**, 432–441 (2023). 37914932 10.1038/s41586-023-06682-5 PMC10632147

[223] Ye, X. et al. Distinct EMT programs control normal mammary stem cells and tumour-initiating cells. *Nature* **525**, 256–260 (2015). 26331542 10.1038/nature14897 PMC4764075

[224] Jiang, H. et al. Chromatin remodeling factor ARID2 suppresses hepatocellular carcinoma metastasis via DNMT1-Snail axis. *Proc. Natl. Acad. Sci. USA* **117**, 4770–4780 (2020). 32071245 10.1073/pnas.1914937117 PMC7060681

[225] Pistore, C. et al. DNA methylation variations are required for epithelial-to-mesenchymal transition induced by cancer-associated fibroblasts in prostate cancer cells. *Oncogene* **36**, 5551–5566 (2017). 28581528 10.1038/onc.2017.159

[226] Zeng, Y. et al. Regulation of EZH2 by SMYD2-mediated lysine methylation is implicated in tumorigenesis. *Cell Rep.* **29**, 1482–1498.e1484 (2019). 31693890 10.1016/j.celrep.2019.10.004

[227] Battistelli, C. et al. The Snail repressor recruits EZH2 to specific genomic sites through the enrollment of the lncRNA HOTAIR in epithelial-to-mesenchymal transition. *Oncogene* **36**, 942–955 (2017). 27452518 10.1038/onc.2016.260 PMC5318668

[228] Hu, Y. et al. G9a and histone deacetylases are crucial for Snail2-mediated E-cadherin repression and metastasis in hepatocellular carcinoma. *Cancer Sci.* **110**, 3442–3452 (2019). 31432592 10.1111/cas.14173 PMC6825017

[229] Punzi, S. et al. WDR5 inhibition halts metastasis dissemination by repressing the mesenchymal phenotype of breast cancer cells. *Breast Cancer Res.* **21**, 123 (2019). 31752957 10.1186/s13058-019-1216-y PMC6873410

[230] Serresi, M. et al. Polycomb repressive complex 2 is a barrier to KRAS-driven inflammation and epithelial-mesenchymal transition in non-small-cell lung cancer. *Cancer Cell* **29**, 17–31 (2016). 26766588 10.1016/j.ccell.2015.12.006

[231] Wanna-Udom, S. et al. KDM2B is involved in the epigenetic regulation of TGF-β-induced epithelial-mesenchymal transition in lung and pancreatic cancer cell lines. *J. Biol. Chem.* **296**, 100213 (2021). 33779563 10.1074/jbc.RA120.015502 PMC7948487

[232] Chaffer, C. L. et al. Poised chromatin at the ZEB1 promoter enables breast cancer cell plasticity and enhances tumorigenicity. *Cell* **154**, 61–74 (2013). 23827675 10.1016/j.cell.2013.06.005 PMC4015106

[233] Sahu, S. K. et al. A complex epigenome-splicing crosstalk governs epithelial-to-mesenchymal transition in metastasis and brain development. *Nat. Cell Biol.* **24**, 1265–1277 (2022). 35941369 10.1038/s41556-022-00971-3

[234] Parreno, V. et al. Transient loss of polycomb components induces an epigenetic cancer fate. *Nature* **629**, 688–696 (2024). 38658752 10.1038/s41586-024-07328-w PMC11096130

[235] Haerinck, J., Goossens, S. & Berx, G. The epithelial-mesenchymal plasticity landscape: principles of design and mechanisms of regulation. *Nat. Rev. Genet.* **24**, 590–609 (2023). 37169858 10.1038/s41576-023-00601-0

[236] Lüönd, F. et al. Distinct contributions of partial and full EMT to breast cancer malignancy. *Dev. Cell* **56**, 3203–3221.e3211 (2021). 34847378 10.1016/j.devcel.2021.11.006

[237] Pastushenko, I. et al. Identification of the tumour transition states occurring during EMT. *Nature* **556**, 463–468 (2018). 29670281 10.1038/s41586-018-0040-3

[238] Simeonov, K. P. et al. Single-cell lineage tracing of metastatic cancer reveals selection of hybrid EMT states. *Cancer Cell* **39**, 1150–1162.e1159 (2021). 34115987 10.1016/j.ccell.2021.05.005 PMC8782207

[239] Bierie, B. et al. Integrin-β4 identifies cancer stem cell-enriched populations of partially mesenchymal carcinoma cells. *Proc. Natl. Acad. Sci. USA* **114**, E2337–e2346 (2017). 28270621 10.1073/pnas.1618298114 PMC5373369

[240] Kröger, C. et al. Acquisition of a hybrid E/M state is essential for tumorigenicity of basal breast cancer cells. *Proc. Natl. Acad. Sci. USA* **116**, 7353–7362 (2019). 30910979 10.1073/pnas.1812876116 PMC6462070

[241] Pastushenko, I. et al. Fat1 deletion promotes hybrid EMT state, tumour stemness and metastasis. *Nature* **589**, 448–455 (2021). 33328637 10.1038/s41586-020-03046-1 PMC7612440

[242] Sample, R. A., Nogueira, M. F., Mitra, R. D. & Puram, S. V. Epigenetic regulation of hybrid epithelial-mesenchymal cell states in cancer. *Oncogene* **42**, 2237–2248 (2023). 37344626 10.1038/s41388-023-02749-9 PMC10578205

[243] Jakab, M. et al. Lung endothelium exploits susceptible tumor cell states to instruct metastatic latency. *Nat. Cancer* **5**, 716–730 (2024). 38308117 10.1038/s43018-023-00716-7 PMC11136671

[244] Gallardo, A. et al. EZH2 represses mesenchymal genes and upholds the epithelial state of breast carcinoma cells. *Cell Death Dis.* **15**, 609 (2024). 39174513 10.1038/s41419-024-07011-y PMC11341823

[245] Gallardo, A. et al. EZH2 endorses cell plasticity to non-small cell lung cancer cells facilitating mesenchymal to epithelial transition and tumour colonization. *Oncogene* **41**, 3611–3624 (2022). 35680984 10.1038/s41388-022-02375-x

[246] Zhang, Y. et al. Genome-wide CRISPR screen identifies PRC2 and KMT2D-COMPASS as regulators of distinct EMT trajectories that contribute differentially to metastasis. *Nat. Cell Biol.* **24**, 554–564 (2022). 35411083 10.1038/s41556-022-00877-0 PMC9037576

[247] Zheng, H. et al. Single-cell analysis reveals cancer stem cell heterogeneity in hepatocellular carcinoma. *Hepatology* **68**, 127–140 (2018). 29315726 10.1002/hep.29778 PMC6033650

[248] Zhou, L. et al. Lineage tracing and single-cell analysis reveal proliferative Prom1+ tumour-propagating cells and their dynamic cellular transition during liver cancer progression. *Gut* **71**, 1656–1668 (2022). 34588223 10.1136/gutjnl-2021-324321

[249] Liu, S. et al. Breast cancer stem cells transition between epithelial and mesenchymal states reflective of their normal counterparts. *Stem Cell Rep.* **2**, 78–91 (2014). 10.1016/j.stemcr.2013.11.009 PMC3916760 24511467

[250] Guilhamon, P. et al. Single-cell chromatin accessibility profiling of glioblastoma identifies an invasive cancer stem cell population associated with lower survival. *Elife* **10**, e64090 (2021). 33427645 10.7554/eLife.64090 PMC7847307

[251] Patel, A. P. et al. Single-cell RNA-seq highlights intratumoral heterogeneity in primary glioblastoma. *Science* **344**, 1396–1401 (2014). 24925914 10.1126/science.1254257 PMC4123637

[252] Gangoso, E. et al. Glioblastomas acquire myeloid-affiliated transcriptional programs via epigenetic immunoediting to elicit immune evasion. *Cell* **184**, 2454–2470.e2426 (2021). 33857425 10.1016/j.cell.2021.03.023 PMC8099351

[253] Muthukrishnan, S. D. et al. P300 promotes tumor recurrence by regulating radiation-induced conversion of glioma stem cells to vascular-like cells. *Nat. Commun.* **13**, 6202 (2022). 36261421 10.1038/s41467-022-33943-0 PMC9582000

[254] Becker, W. R. et al. Single-cell analyses define a continuum of cell state and composition changes in the malignant transformation of polyps to colorectal cancer. *Nat. Genet* **54**, 985–995 (2022). 35726067 10.1038/s41588-022-01088-x PMC9279149

[255] Lenos, K. J. et al. Stem cell functionality is microenvironmentally defined during tumour expansion and therapy response in colon cancer. *Nat. Cell Biol.* **20**, 1193–1202 (2018). 30177776 10.1038/s41556-018-0179-z PMC6163039

[256] Álvarez-Varela, A. et al. Mex3a marks drug-tolerant persister colorectal cancer cells that mediate relapse after chemotherapy. *Nat. Cancer* **3**, 1052–1070 (2022). 35773527 10.1038/s43018-022-00402-0

[257] Kamachi, K. et al. Targeting DNMT1 by demethylating agent OR-2100 increases tyrosine kinase inhibitors-sensitivity and depletes leukemic stem cells in chronic myeloid leukemia. *Cancer Lett.* **526**, 273–283 (2022). 34875342 10.1016/j.canlet.2021.11.032

[258] Saunthararajah, Y. et al. Evaluation of noncytotoxic DNMT1-depleting therapy in patients with myelodysplastic syndromes. *J. Clin. Investig.* **125**, 1043–1055 (2015). 25621498 10.1172/JCI78789 PMC4362268

[259] Pathania, R. et al. Combined inhibition of DNMT and HDAC blocks the tumorigenicity of cancer stem-like cells and attenuates mammary tumor growth. *Cancer Res.* **76**, 3224–3235 (2016). 27197203 10.1158/0008-5472.CAN-15-2249 PMC4891240

[260] Zavras, P. D. et al. Clinical trials assessing hypomethylating agents combined with other therapies: causes for failure and potential solutions. *Clin. Cancer Res.* **27**, 6653–6661 (2021). 34551907 10.1158/1078-0432.CCR-21-2139 PMC8678301

[261] Wang, F. et al. Leukemia stemness and co-occurring mutations drive resistance to IDH inhibitors in acute myeloid leukemia. *Nat. Commun.* **12**, 2607 (2021). 33972549 10.1038/s41467-021-22874-x PMC8110775

[262] Turcan, S. et al. Mutant-IDH1-dependent chromatin state reprogramming, reversibility, and persistence. *Nat. Genet.* **50**, 62–72 (2018). 29180699 10.1038/s41588-017-0001-z PMC5769471

[263] Guryanova, O. A. et al. DNMT3A mutations promote anthracycline resistance in acute myeloid leukemia via impaired nucleosome remodeling. *Nat. Med.* **22**, 1488–1495 (2016). 27841873 10.1038/nm.4210 PMC5359771

[264] Ramabadran, R. et al. DNMT3A-coordinated splicing governs the stem state switch towards differentiation in embryonic and haematopoietic stem cells. *Nat. Cell Biol.* **25**, 528–539 (2023). 37024683 10.1038/s41556-023-01109-9 PMC10337578

[265] Hu, B. et al. CD13 promotes hepatocellular carcinogenesis and sorafenib resistance by activating HDAC5-LSD1-NF-κB oncogenic signaling. *Clin. Transl. Med.* **10**, e233 (2020). 33377659 10.1002/ctm2.233 PMC7708822

[266] Huang, M. et al. Targeting KDM1A attenuates Wnt/β-catenin signaling pathway to eliminate sorafenib-resistant stem-like cells in hepatocellular carcinoma. *Cancer Lett.* **398**, 12–21 (2017). 28377178 10.1016/j.canlet.2017.03.038

[267] Galluzzi, L. et al. Targeting immunogenic cell stress and death for cancer therapy. *Nat. Rev. Drug Discov.* **23**, 445–460 (2024). 38622310 10.1038/s41573-024-00920-9 PMC11153000

[268] Marchi, S. et al. Mitochondrial control of inflammation. *Nat. Rev. Immunol.* **23**, 159–173 (2023). 35879417 10.1038/s41577-022-00760-x PMC9310369

[269] Luo, C. W. et al. G9a governs colon cancer stem cell phenotype and chemoradioresistance through PP2A-RPA axis-mediated DNA damage response. *Radiother. Oncol.* **124**, 395–402 (2017). 28351524 10.1016/j.radonc.2017.03.002

[270] Rowbotham, S. P. et al. H3K9 methyltransferases and demethylases control lung tumor-propagating cells and lung cancer progression. *Nat. Commun.* **9**, 4559 (2018). 30455465 10.1038/s41467-018-07077-1 PMC6242814

[271] Avgustinova, A. et al. Loss of G9a preserves mutation patterns but increases chromatin accessibility, genomic instability and aggressiveness in skin tumours. *Nat. Cell Biol.* **20**, 1400–1409 (2018). 30455462 10.1038/s41556-018-0233-x

[272] Nachiyappan, A. et al. EHMT1 promotes tumor progression and maintains stemness by regulating ALDH1A1 expression in alveolar rhabdomyosarcoma. *J. Pathol.* **256**, 349–362 (2022). 34897678 10.1002/path.5848

[273] Metzger, E. et al. KDM4 inhibition targets breast cancer stem-like cells. *Cancer Res.* **77**, 5900–5912 (2017). 28883001 10.1158/0008-5472.CAN-17-1754

[274] Zhou, J. et al. PTEN is fundamental for elimination of leukemia stem cells mediated by GSK126 targeting EZH2 in chronic myelogenous leukemia. *Clin. Cancer Res.* **24**, 145–157 (2018). 29070525 10.1158/1078-0432.CCR-17-1533

[275] Göllner, S. et al. Loss of the histone methyltransferase EZH2 induces resistance to multiple drugs in acute myeloid leukemia. *Nat. Med.* **23**, 69–78 (2017). 27941792 10.1038/nm.4247 PMC6548550

[276] Celik, H. et al. JARID2 functions as a tumor suppressor in myeloid neoplasms by repressing self-renewal in hematopoietic progenitor cells. *Cancer Cell* **34**, 741–756.e748 (2018). 30423295 10.1016/j.ccell.2018.10.008 PMC6237100

[277] Brien, G. L. et al. Simultaneous disruption of PRC2 and enhancer function underlies histone H3.3-K27M oncogenic activity in human hindbrain neural stem cells. *Nat. Genet.* **53**, 1221–1232 (2021). 34294917 10.1038/s41588-021-00897-w

[278] Lund, K., Adams, P. D. & Copland, M. EZH2 in normal and malignant hematopoiesis. *Leukemia* **28**, 44–49 (2014). 24097338 10.1038/leu.2013.288

[279] Wassef, M. et al. EZH1/2 function mostly within canonical PRC2 and exhibit proliferation-dependent redundancy that shapes mutational signatures in cancer. *Proc. Natl. Acad. Sci. USA* **116**, 6075–6080 (2019). 30867289 10.1073/pnas.1814634116 PMC6442582

[280] Liu, Y. W. et al. LincHOTAIR epigenetically silences miR34a by binding to PRC2 to promote the epithelial-to-mesenchymal transition in human gastric cancer. *Cell Death Dis.* **6**, e1802 (2015). 26136075 10.1038/cddis.2015.150 PMC4650715

[281] Shi, T. H., Sugishita, H. & Gotoh, Y. Crosstalk within and beyond the polycomb repressive system. *J. Cell Biol.* **223**, e202311021 (2024). 38506728 10.1083/jcb.202311021 PMC10955045

[282] Huang, J. et al. The noncanonical role of EZH2 in cancer. *Cancer Sci.* **112**, 1376–1382 (2021). 33615636 10.1111/cas.14840 PMC8019201

[283] Gozdecka, M. et al. UTX-mediated enhancer and chromatin remodeling suppresses myeloid leukemogenesis through noncatalytic inverse regulation of ETS and GATA programs. *Nat. Genet.* **50**, 883–894 (2018). 29736013 10.1038/s41588-018-0114-z PMC6029661

[284] Stief, S. M. et al. Loss of KDM6A confers drug resistance in acute myeloid leukemia. *Leukemia* **34**, 50–62 (2020). 31201358 10.1038/s41375-019-0497-6 PMC7214274

[285] Ho, L. L. et al. DOT1L-mediated H3K79 methylation in chromatin is dispensable for Wnt pathway-specific and other intestinal epithelial functions. *Mol. Cell Biol.* **33**, 1735–1745 (2013). 23428873 10.1128/MCB.01463-12 PMC3624170

[286] He, J., Nguyen, A. T. & Zhang, Y. KDM2b/JHDM1b, an H3K36me2-specific demethylase, is required for initiation and maintenance of acute myeloid leukemia. *Blood* **117**, 3869–3880 (2011). 21310926 10.1182/blood-2010-10-312736 PMC3083299

[287] Wu, Q. et al. PRMT inhibition induces a viral mimicry response in triple-negative breast cancer. *Nat. Chem. Biol.* **18**, 821–830 (2022). 35578032 10.1038/s41589-022-01024-4 PMC9337992

[288] Sachamitr, P. et al. PRMT5 inhibition disrupts splicing and stemness in glioblastoma. *Nat. Commun.* **12**, 979 (2021). 33579912 10.1038/s41467-021-21204-5 PMC7881162

[289] Rondeau, V. et al. Spermidine metabolism regulates leukemia stem and progenitor cell function through KAT7 expression in patient-derived mouse models. *Sci. Transl. Med* **16**, eadn1285 (2024). 39321266 10.1126/scitranslmed.adn1285 PMC11670652

[290] Nguyen, M. U. et al. KAT2A and KAT2B prevent double-stranded RNA accumulation and interferon signaling to maintain intestinal stem cell renewal. *Sci. Adv.* **10**, eadl1584 (2024). 39110797 10.1126/sciadv.adl1584 PMC11305398

[291] Chen, D. et al. Targeting BMI1(+) cancer stem cells overcomes chemoresistance and inhibits metastases in squamous cell carcinoma. *Cell Stem Cell* **20**, 621–634.e626 (2017). 28285905 10.1016/j.stem.2017.02.003 PMC5419860

[292] Bansal, N. et al. BMI-1 targeting interferes with patient-derived tumor-initiating cell survival and tumor growth in prostate cancer. *Clin. Cancer Res.* **22**, 6176–6191 (2016). 27307599 10.1158/1078-0432.CCR-15-3107 PMC5159329

[293] Zhao, S., Allis, C. D. & Wang, G. G. The language of chromatin modification in human cancers. *Nat. Rev. Cancer* **21**, 413–430 (2021). 34002060 10.1038/s41568-021-00357-x PMC10507815

[294] Clapier, C. R., Iwasa, J., Cairns, B. R. & Peterson, C. L. Mechanisms of action and regulation of ATP-dependent chromatin-remodelling complexes. *Nat. Rev. Mol. Cell Biol.* **18**, 407–422 (2017). 28512350 10.1038/nrm.2017.26 PMC8127953

[295] Delaunay, S., Helm, M. & Frye, M. RNA modifications in physiology and disease: towards clinical applications. *Nat. Rev. Genet.* **25**, 104–122 (2024). 37714958 10.1038/s41576-023-00645-2

[296] Kan, R. L., Chen, J. & Sallam, T. Crosstalk between epitranscriptomic and epigenetic mechanisms in gene regulation. *Trends Genet.* **38**, 182–193 (2022). 34294427 10.1016/j.tig.2021.06.014 PMC9093201

[297] Louwagie, A. & Vu, L. P. Emerging interactions between RNA methylation and chromatin architecture. *Curr. Opin. Genet. Dev.* **89**, 102270 (2024). 39426116 10.1016/j.gde.2024.102270

[298] Wang, Y., Huang, H., Chen, J. & Weng, H. Crosstalk between histone/DNA modifications and RNA N(6)-methyladenosine modification. *Curr. Opin. Genet. Dev.* **86**, 102205 (2024). 38776766 10.1016/j.gde.2024.102205

[299] Chen, X. et al. METTL14-mediated N6-methyladenosine modification of SOX4 mRNA inhibits tumor metastasis in colorectal cancer. *Mol. Cancer* **19**, 106 (2020). 32552762 10.1186/s12943-020-01220-7 PMC7298962

[300] Zhang, J. et al. Excessive miR-25-3p maturation via N(6)-methyladenosine stimulated by cigarette smoke promotes pancreatic cancer progression. *Nat. Commun.* **10**, 1858 (2019). 31015415 10.1038/s41467-019-09712-x PMC6478927

[301] Gupta, R. A. et al. Long non-coding RNA HOTAIR reprograms chromatin state to promote cancer metastasis. *Nature* **464**, 1071–1076 (2010). 20393566 10.1038/nature08975 PMC3049919

[302] Sun, M. et al. LncRNA HOXA11-AS promotes proliferation and invasion of gastric cancer by scaffolding the chromatin modification factors PRC2, LSD1, and DNMT1. *Cancer Res.* **76**, 6299–6310 (2016). 27651312 10.1158/0008-5472.CAN-16-0356

[303] Li, Z. et al. The degradation of EZH2 mediated by lncRNA ANCR attenuated the invasion and metastasis of breast cancer. *Cell Death Differ.* **24**, 59–71 (2017). 27716745 10.1038/cdd.2016.95 PMC5260507

[304] Luo, J. et al. LncRNA-p21 alters the antiandrogen enzalutamide-induced prostate cancer neuroendocrine differentiation via modulating the EZH2/STAT3 signaling. *Nat. Commun.* **10**, 2571 (2019). 31189930 10.1038/s41467-019-09784-9 PMC6561926

[305] Guo, K. et al. LncRNA-MIAT promotes thyroid cancer progression and function as ceRNA to target EZH2 by sponging miR-150-5p. *Cell Death Dis.* **12**, 1097 (2021). 34811354 10.1038/s41419-021-04386-0 PMC8608816

[306] Wang, H. et al. STAT3-mediated upregulation of lncRNA HOXD-AS1 as a ceRNA facilitates liver cancer metastasis by regulating SOX4. *Mol. Cancer* **16**, 136 (2017). 28810927 10.1186/s12943-017-0680-1 PMC5558651

